# Mitochondrial genome evolution and tRNA truncation in Acariformes mites: new evidence from eriophyoid mites

**DOI:** 10.1038/srep18920

**Published:** 2016-01-06

**Authors:** Xiao-Feng Xue, Jing-Feng Guo, Yan Dong, Xiao-Yue Hong, Renfu Shao

**Affiliations:** 1 Department of Entomology, Nanjing Agricultural University, Nanjing, Jiangsu 210095, China; 2 GeneCology Research Centre, Faculty of Science, Health, Education and Engineering, University of the Sunshine Coast, Maroochydore, Queensland 4556, Australia

## Abstract

The subclass Acari (mites and ticks) comprises two super-orders: Acariformes and Parasitiformes. Most species of the Parasitiformes known retained the ancestral pattern of mitochondrial (mt) gene arrangement of arthropods, and their mt tRNAs have the typical cloverleaf structure. All of the species of the Acariformes known, however, have rearranged mt genomes and truncated mt tRNAs. We sequenced the mt genomes of two species of Eriophyoidea: *Phyllocoptes taishanensis* and *Epitrimerus sabinae*. The mt genomes of *P. taishanensis* and *E. sabinae* are 13,475 bp and 13,531 bp, respectively, are circular and contain the 37 genes typical of animals; most mt tRNAs are highly truncated in both mites. On the other hand, these two eriophyoid mites have the least rearranged mt genomes seen in the Acariformes. Comparison between eriophyoid mites and other Aacariformes mites showed that: 1) the most recent common ancestor of Acariformes mites retained the ancestral pattern of mt gene arrangement of arthropods with slight modifications; 2) truncation of tRNAs for cysteine, phenylalanine and histidine occurred once in the most recent common ancestor of Acariformes mites whereas truncation of other tRNAs occurred multiple times; and 3) the placement of eriophyoid mites in the order Trombidiformes needs to be reviewed.

Eriophyoidea is the largest superfamily in the subclass Acari (mites and ticks), with more than 4,400 described species^1^. Eriophyoid mites have exceptional morphological and biological characters in comparison to other mites and ticks: two pairs of legs (instead of four pairs), small body size (~200 μm body length), and high host specificity^2^, to name a few. Some species of eriophyoid mites are pests to crops^3^ and forests^4^, such as the wheat curl mite, *Aceria tosichella*^5^,^6^. Other than causing yield loss, some eriophyoid mites can transmit and spread viruses, which can cause further damages to plants^6^,^7^.

The typical animal mt genome is circular, 15–20 kb in length with 37 genes^8^, and was found in most mites and ticks investigated to date except for *Steganacarus magnus* (16 tRNA genes not identified, possibly due to tRNA truncation)^9^, *Metaseiulus occidentalis* (*nad3* and *nad6* not identified, 18 genes duplicated)^10^ and *Leptotrombidium pallidum* (2 genes duplicated)^11^. Of the 39 species from the superorder Parasitiformes investigated to date, 19 species retained the ancestral pattern of mt gene arrangement for arthropods; these species are from four families: Argasidae (soft ticks, 11 species)^12^,^13^,^14^, Allothyridae (holothyroid mites, 1 species)^12^, Nuttalliellidae (hard ticks, 1 species)^13^ and Ixodidae (hard ticks, 6 species)^15^,^16^,^17^. Rearrangement of mt genes was found in the other 20 species from five families: Ixodidae (16 species)^18^,^19^, Varroidae (1 species)^20^, Phytoseiidae (2 species)^10^,^21^ and Ologamasidae (1 species)^22^. In contrast to Parasitiformes, all of the 27 species from the other superorder, Acariformes, have rearranged or highly rearranged mt genomes (details below).

The mt tRNAs of animals usually possess a cloverleaf secondary structure composed of four arms: AA-arm, D-arm, AC-arm and T-arm^23^. The only exception is the tRNA for serine (anticodon GCT), which lost D-arm in nearly all animals; this is apparently an ancestral feature for animals^23^. Loss of D-arm or T-arm occurred in other tRNAs but was not common in animals. Large-scale tRNA truncation was first found in nematodes, in which 20 of the 22 tRNAs lack T-arm and the two tRNAs for serine (anticodons GCT and TGA) lack the D-arm^24^. Later, truncated tRNAs were also found in Acariformes^11^, Araneae^25^,^26^,^27^, Pseudoscorpiones^28^, Scorpiones^29^,^30^, Thelyphonida^30^, Acanthocephala^31^, Insecta^32^ and Protura^33^. All of the 25 species of Acariformes mites known have many truncated tRNA genes whereas species from the other superorder, Parasitiformes, do not have truncated tRNAs except for the tRNA for serine (anticodon GCT), and the tRNA for cysteine, which lacks D-arm in *Varroa destructor*^20^, *Phytoseiulus persimilis*^21^, *M. occidentalis*^21^, *Haemaphysalis flava*^14^, *Rhipicephalus microplus*^14^ and *R. sanguineus*^14^.

Prior to this study, no complete mt genomes have been investigated for eriophyoid mites. To understand the evolution of mt genomes in the Acariformes, we sequenced the mt genomes of two eriophyoid mites, *Phyllocoptes taishanensis* Xue & Hong, 2005 (Eriophyidae: Phyllocoptini) and *Epitrimerus sabinae* Xue & Hong, 2005 (Eriophyidae: Phyllocoptini). We found rearrangement of mt genes in both eriophyoid mites relative to the hypothetical ancestor of arthropods. Further, both species have highly truncated tRNAs. Here, we present the novel features of the mt genomes of *P. taishanensis* and *E. sabinae* and discuss the evolution of mt genomes in the Acariformes, tRNA truncation and the phylogeny of eriophyoid mites in the light of new evidence from these two mites.

## Results

### General features of the mt genomes of the two eriophyoid mites, *P*. *taishanensis* and *E*. *sabinae*

**Figure 1:**
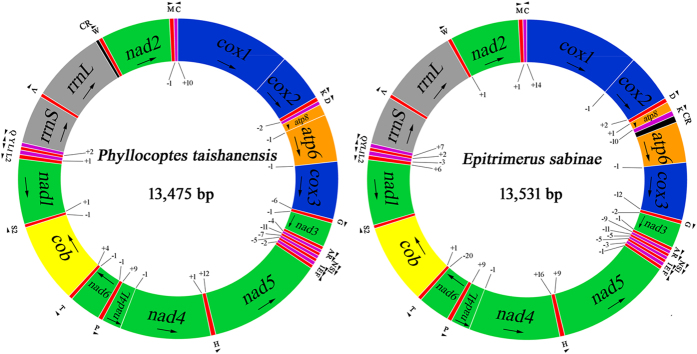
Map of the mitochondrial genomes of *Phyllocoptes taishanensis* (A) and *Epitrimerus sabinae* (B). Protein-coding genes are color-coded (cox: blue; nad: green; atp: orange; cob: yellow); rRNA genes are in grey; control regions are in black; tRNA genes are in red or purple. Abbreviations of protein-coding genes are: *atp6* and *atp8* for ATP synthase subunits 6 and 8, *cox1–3* for cytochrome oxidase subunits 1–3, *cob* for cytochrome b, *nad1-6* and *nad4L* for NADH dehydrogenase subunits 1-6 and 4 L, *rrnL* and *rrnS* for large and small rRNA subunits. tRNA genes are indicated by the single letter IUPAC-IUB abbreviations for their corresponding amino acids. Arrows and arrowheads show the direction of gene transcription. Numbers at gene junctions indicate the length of non-coding sequences; negative numbers indicate overlap between genes.

The mt genomes of *P. taishanensis* and *E. sabinae* are 13, 475 bp and 13, 531 bp long respectively, are circular and have 37 genes: 13 protein-coding genes (PCG), two ribosomal RNA (rRNA) genes and 22 transfer RNA (tRNA) genes (Fig. 1; Table 1). Genes are on both strands: one strand has 27 genes whereas the other strand has 10 genes. The start codons of the 13 PCGs were ATN in *E. sabinae*. In *P. taishanensis*, 11 of the 13 PCGs use ATN as start codons whereas *cox1* and *atp8* appear to start with CTG, which is a rare start codon for animal mitochondria (http://www.ncbi.nlm.nih.gov/Taxonomy/taxonomyhome.html/index.cgi ? chapter=cgencodes). We noticed that in the water mites, *Unionicola parkeri* and *U. foili*, another rare start codon, TTG, was used^34^,^35^. The stop codons were TAA or TAG in both eriophyoid species; incomplete stop codons, T, was found in protein-coding genes that precede a tRNA gene. In both species of eriophyoid mites, TAA was the most common stop codon and was used in nine of the 13 PCGs in *P. taishanensis* and 10 of the 13 PCGs in *E. sabinae* (Table 1). The putative control region (CR) of *P*. *taishanensis* is short, only 47 bp in size, lying between *rrnL* and *trnW*. The putative CR of *E*. *sabinae* is 94 bp and is in a different location between *trnK* and *atp6*. No conserved regions were found between the CRs of the two eriophyoid mites. No other non-coding regions longer than 16 bp were found in the mt genomes of these two mites.

### Eriophyoid mites have the least rearranged mt genomes among Acariformes mites

**Figure 2:**
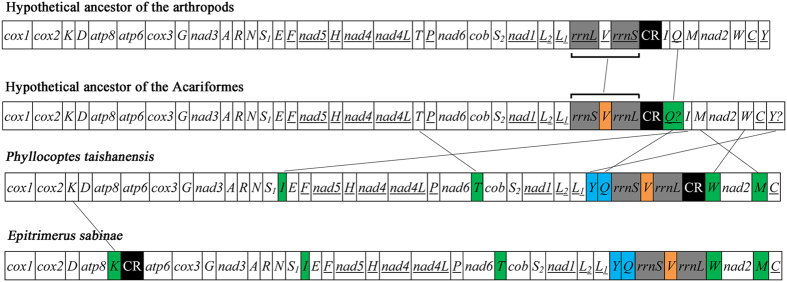
Mitochondrial gene arrangements in the hypothetical ancestor of Acariformes, *Phyllocoptes taishanensis* and *Epitrimerus sabinae*. Underlined genes are on the N-strand. Translocated or inverted genes are color-coded (blue: inversion and translocation; green: translocation; orange: inversion). rRNA genes are in grey; control regions are in black. Abbreviations of gene names are the same as in Fig. 1. Lines between genes indicate gene rearrangement. The positions of *trnQ* and *trnY* are unclear. Question marks after tRNA genes indicate uncertain locations.

Like in other mites of the Acariformes, rearrangement of mt genes occurred in both of the eriophyoid mites. We calculated breakpoints with CREx as a measure of the extent of mt gene rearrangement from that of the hypothetical ancestor of arthropods^36^. Among the 12 different patterns of mt gene arrangement observed in the 27 species of Acariformes mites known, *P*. *taishanensis* has the least rearranged mt genome with 13 breakpoints and *E*. *sabinae* has the third least rearranged mt genome with 16 breakpoints (Table 2). In *P. taishanensis*, two rRNA genes (*rrnS* and *rrnL*) and seven tRNA genes (*trnI*, *trnT*, *trnY*, *trnQ*, *trnV*, *trnW*, *trnM*) changed their locations relative to their counterpart genes in the hypothetical ancestor of arthropods. In *E. sabinae*, two rRNA genes (*rrnS* and *rrnL*) and eight tRNA genes (*trnK*, *trnI*, *trnT*, *trnY*, *trnQ*, *trnV*, *trnW*, *trnM*) changed locations (Fig. 2). Four derived gene arrangements, *trnS*_*1*_-*trnI*-*trnE*, *nad6*-*trnT*-*cob*, *trnY*-*trnQ*-*rrnS*-*trnV*-*rrnL*, and *trnW*-*nad2*-*trnM-trnC*, were found in both eriophyoid mites but not in any other mites; these novel gene arrangements were candidate synapomorphies (i.e. shared derived characters) for the eriophyoid mites.

### Truncated tRNAs of the eriophyoid mites

**Figure 3:**
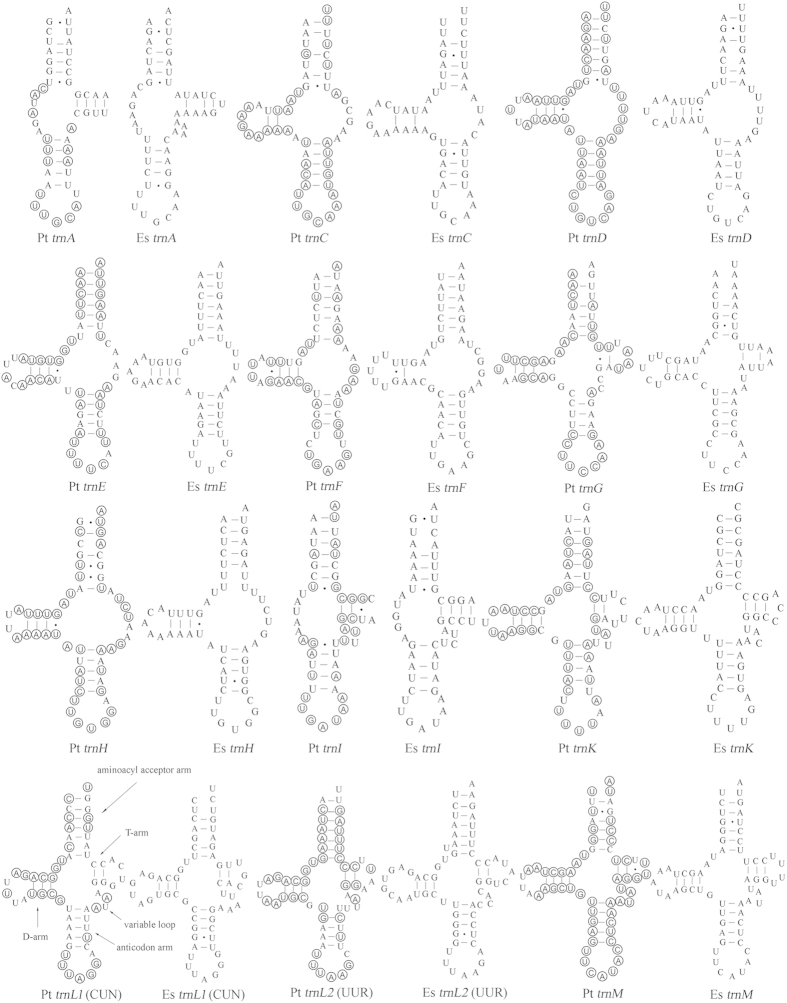
Inferred secondary structures of the 12 mt tRNAs of *P. taishanensis* (Pt) and *E. sabinae* (Es). tRNAs are labeled with the abbreviations of their corresponding amino acids. Dashes indicate Watson–Crick bonds; dots indicate bonds between U and G. Shared identical sequences between tRNA genes are circled in *P. taishanensis*.

**Figure 4:**
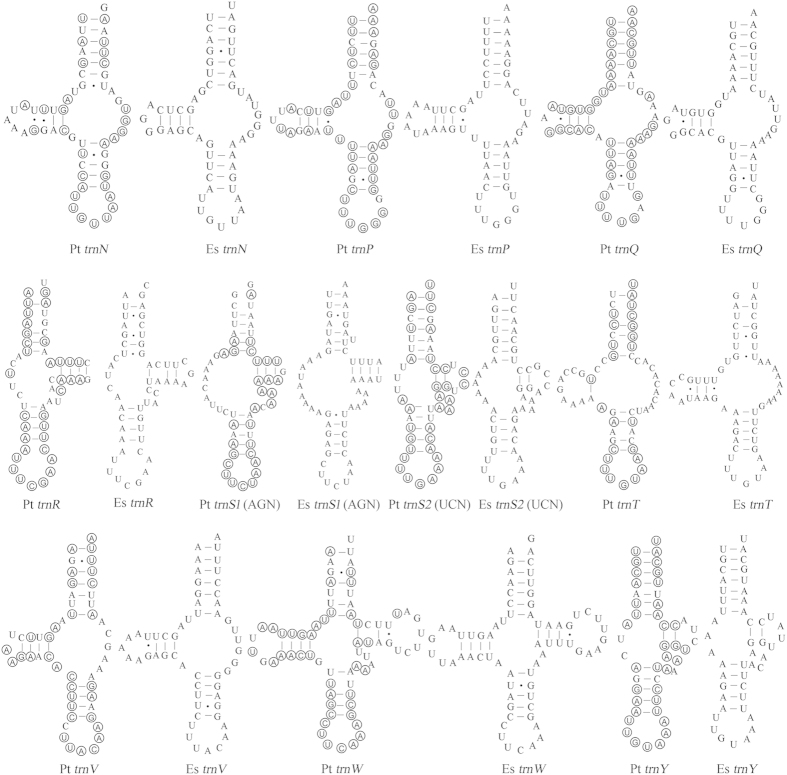
Inferred secondary structures of the 10 mt tRNAs of *P. taishanensis* (Pt) and *E. sabinae* (Es). tRNAs are labeled with the abbreviations of their corresponding amino acids. Dashes indicate Watson–Crick bonds; dots indicate bonds between U and G. Shared identical sequences between tRNA genes are circled in *P. taishanensis*.

Fifteen mt tRNA genes of *P. taishanensis* and 16 mt tRNA genes of *E. sabinae* were identified with tRNAscan-SE^37^ or ARWEN^38^ programs. The other seven tRNA genes of *P. taishanensis* (*trnA*, *trnG*, *trnQ*, *trnR*, *trnS*_*1*_, *trnT*, *trnV*) and the other six tRNA genes of *E. sabinae* (*trnA*, *trnG*, *trnI*, *trnQ*, *trnR*, *trnS*_*1*_) were found manually based on conserved nucleotides and the anticodon sequences. The putative mt tRNAs were highly truncated in both *P*. *taishanensis* (47 to 61 bp) and *E*. *sabinae* (47 to 67 bp) (Figs 3 and 4). Sixteen of the 22 tRNAs have atypical secondary structures, missing either D-arm or T-arm in both mites. The majority of tRNAs also have mismatches on T-arm, D-arm, acceptor arm or anticodon arm.

### The phylogenetic position of Eriophyoidea

**Figure 5:**
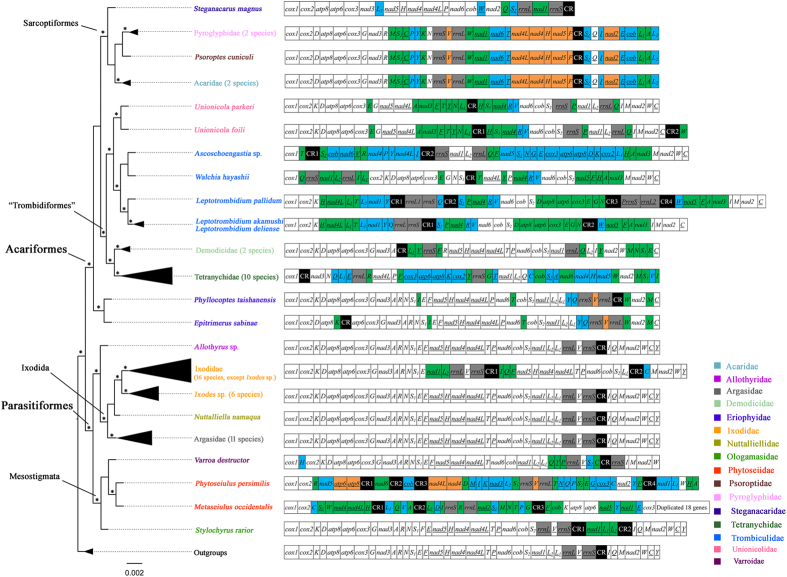
Maximum likelihood trees inferred with nucleotide sequences by 16 partitions (13 PCGs, 2 rRNA genes and concatenated tRNA genes). Asterisks indicate the branches with >75% BSPs and>95% BPPs in the majority of the 13 topologies (Figures S1 and S2). Translocated genes are in green; inverted genes are in orange; inverted and translocated genes are in blue. rRNAs are in grey. Control regions are in black. Abbreviations of gene names are the same as in Fig. 1. Genes are transcribed from left to right except for those underlined, which are transcribed from right to left.

The super-family Eriophyoidea was traditionally in the order Trombidiformes. In our analysis, however, the two eriophyoid species were not grouped with spider mites (Tetranychidae), follicle mites (Demodicidae), chigger mites (Trombiculidae) and water mites (Unionicolidae), which were in the Trombidiformes. Rather, these two eriophyoid mites were grouped with Sarcoptiformes mites in ML and BI trees with strong support (Figures S1A-S1B, S1F, BSPs =100; Figure S2A, BPPs =100), or at the base in the Acariformes (Fig. 5, Figures S1C-S1E, Figures S2B-S2H). Excluding the two eriophyoid mites, the other Trombidiformes mites formed a monophyletic group with strong support (Table 3; Figures S1A-S1D, BSPs =100; Figures S2A, S2C-S2H, BPPs =100; Figure S2B, BPPs = 58). In addition, our phylogenetic analyses support the monophyly of Acariformes in all ML and BI trees (Table 3; Fig. 5, Figures S1A-S1F, BSPs >97; Figures S2A-S2I, BPPs =100). Monophyly of the other superorder, Parasitiformes, was recovered in ML and BI trees constructed with nucleotide sequences (Table 3; Figure S1A- S1D, S1F, BSPs >98; Figure S2A, S2C-S2I, BPPs =100), but was rejected in ML and BI trees constructed with amino acid sequences (Table 3, Figure S1E, Figure S2B). Also, three orders (Ixodida, Mesostigmata and Sarcoptiformes) were recovered as monophyletic groups in most of our analyses (Table 3, Fig. 5). Nine families (Acaridae, Argasidae, Demodicidae, Eriophyidae, Ixodidae, Phytoseiidae, Tetranychidae, Trombiculidae and Unionicolidae) were recovered as monophyletic groups in all of our analyses (Table 3, BSPs =100, BPPs =100). Intriguingly, monophyly of Acari was rejected as Pseudoscorpiones was grouped with Acariformes with strong support (Figure S1F, BSPs =98; Figure S2I, BPPs = 100). Our result thus conflicts with previous analyses based on rDNA in which Solifugae was grouped with Acariformes^39^,^40^,^41^, indicating the weaknesses of current hypotheses on the ordinal relationships in the Arachnida.

## Discussion

### The ancestral pattern of mt gene arrangement of Acariformes mites

Mitochondrial gene arrangement is relatively stable within major animal lineages^8^. This is, however, not the case for the Acari (ticks and mites). For the superorder Parasitiformes, the ancestral pattern of mt gene arrangement to arthropods was found in 11 species of Argasidae, one species of Allothyridae and six *Ixodes* species of Ixodidae (Fig. 5). Four Mesostigmata species and 16 Ixodidae species have rearrangement of mt genes (Fig. 5, Table 2). In the other superorder Acariformes, however, rearrangement of mt genes was found in all of the 27 species whose mt genomes have been sequenced. Even the species in the same genus differ from one other in mt gene arrangement, such as the two water mites in the genus *Unionicola*^34^,^35^ and three chigger mites in the genus *Leptotrombidium*^11^,^42^ (Fig. 5). The two eriophyoid mites investigated in the current study were from the same tribe (Phyllocoptini) of the family Eriophyidae but differ from one another in mt gene arrangement.

What was the ancestral pattern of mt gene arrangement for Acariformes mites ? Establishment of the ancestral pattern will lay the ground for understanding how mt genome organization evolved in Acariformes mites. Intriguingly, in comparison to other species of the Acariformes, the two eriophyoid mites have the least rearranged mt genomes and retained most of the ancestral pattern of mt gene arrangement of arthropods (Figs 2 and 5). The conserved mt gene-arrangement characters present in the two eriophyoid mites and other Acariformes mites allowed us to infer that the ancestral pattern of mt gene arrangement of the Acariformes retained likely the ancestral pattern of mt gene arrangement of arthropods with slight modifications (Fig. 2). The modifications include: 1) translocations of *trnQ* and *trnY*; these two tRNA genes are rearranged in all of the 27 species of Acariformes mites investigated to date and furthermore, their locations vary among different Acariformes lineages (Figs 2 and 5) inversion of *rrnS-trnV-rrnL* as a cluster, which is seen in the two eriophyoid mites and five Sarcoptiformes mites (Fig. 5); however, in seven of the 27 Acariformes species, *rrnS* and *rrnL* are translocated but not inverted, so there are alternative interpretations for the location and transcription orientation of *rrnS-trnV-rrnL* in the ancestor of Acariformes mites.

### tRNA truncation in Acariformes mites

Truncated mt tRNAs have been found in several orders of the class Arachnida including Araneae^25^,^26^,^27^, Acariformes^11^, Pseudoscorpiones^28^, Scorpiones^29^,^30^, and Thelyphonida^30^. In the superorder Acariformes, truncated tRNAs are common and have been observed in all of the 27 species whose mt genomes have been sequenced. Sixteen of the 22 tRNAs in the two eriophyoid mites investigated in the current study lack D-arm or T-arm (Figs 3 and 4). Nineteen tRNAs lack D-arm or T-arm in spider mites (Tetranychidae); in some species, the tRNAs for phenylalanine and glutamine lack both D-arm and T-arm (Table S1)^43^,^44^. Fifteen tRNAs lost D-arm or T-arm in Demodicidae, including five tRNAs losing both D-arm and T-arm^45^. Twenty tRNAs in Pyroglyphidae^46^,^47^, 17 to 18 tRNAs in Trombiculidae^11^,^34^,^42^, 21 tRNAs in Acaridae and Psoroptidae^48^,^49^,^50^, 15 tRNAs in Unionicolidae^34^,^35^, lack D-arm or T-arm (Table S1).

Large-scale tRNA truncation was first observed, and best studied in nematodes^24^. Twenty of the 22 tRNAs lack T-arm and the two tRNAs for serine lack D-arm in all nematodes except for several species in the class Enoplea. In *Trichinella spiralis*, eight tRNAs have the cloverleaf secondary structure with both D- and T-arms whereas several others lack both D- and T-arms^51^. tRNA truncation in the Acariformes mites has some similarity to that in nematodes but has their distinctive features. First, *trnK* has the typical cloverleaf structure in all Acariformes mites except for *Steganacarus magnus*^9^ (but K was not identified in this mite, see Table S1), whereas the other 21 tRNAs lack either D-arm or T-arm or both arms in one or more species of Acariformes mites. Three tRNAs, for cysteine, phenylalanine and histidine respectively, lack T-arm in all known Acariformes mites (Table S1), indicating that T-arm loss in these three tRNAs is likely ancestral to Acariformes mites. The other 18 tRNAs, however, vary in their secondary structures among the Acariformes mites: either having a cloverleaf structure, or lacking D-arm, T-arm or both arms (Table S1). The pattern of D-arm or T-arm loss appears to be consistent within a family but differs between families (Table S1). Thus, the loss of D-arm or T-arm in these tRNAs may have occurred multiple times independently in different lineages of the Acariformes mites.

Limited evidence from nematodes indicates that truncation does not seem to disable mt tRNAs from function. Okimoto *et al.* hybridized mt tRNA gene-specific probes to the RNAs of two nematodes, *Caenorhabditis elegans* and *Ascaris suum*, and obtained evidence for the transcription of at least nine *C. elegans* and three *A. suum* mt tRNA genes^52^. Each tRNA transcript has the same size of its corresponding tRNA gene, to which three nucleotides (CCA) were added after transcription. Okimoto *et al.* concluded that the mt tRNAs without D-arm or T-arm were functional. Further, Suematsu *et al.* and Ohtsuki and Watanabe showed that the evolution of truncated mt tRNAs in nematodes was linked to EF-Tu protein: the paralogs of this protein acquired differential binding abilities to tRNAs with deleted domains^53^,^54^. Recently, Juhling *et al.* showed computational evidence that tRNAs of the nematodes of the class Enoplea that lack both D-arm and T-arm were functional^51^. The Acariformes mites provided another system for further experimental and computational investigation into the evolution and function of truncated mt tRNAs.

### Should Eriophyoidea be placed in the order Trombidiformes?

The subclass Acari comprises two superorders, Acariformes and Parasitiformes^55^. The former includes two orders (Trombidiformes and Sarcoptiformes), while the later include four orders (Opilioacarida, Holothyrida, Ixodida, Mesostigmata)^55^. The monophyly of Acari, however, is not without controversy, when species of other major lineages of Arachnida were included into analyses^28^,^40^,^41^,^56^. Pseudoscorpiones was always grouped with Acariformes in our analyses, which is consistent with Ovchinnikov and Masta^28^. Several phylogenetic studies of Acariformes^39^,^40^,^41^ and Parasitiformes^57^ have been conducted previously using molecular data; however, eriophyoid mites were not included. The current taxonomic assignment of Eriophyoidea to the order Trombidiformes^55^ is controversial, due to the distinctive morphology of eriophyoid mites such as having only two pairs of legs, lack of ontogenetic diversity and absence of respirator system^58^. Indeed, André placed Eriophyoidea outside Trombidiformes^59^ Our analyses based on different partitions and inference methods showed consistently that Acariformes, Parasitiformes and several families (Acaridae, Argasidae, Demodicidae, Ixodidae, Phytoseiidae, Tetranychidae, Trombiculidae and Unionicolidae) were monophyletic, which was consistent with previous studies using mt genes^9^,^18^,^27^,^39^,^44^,^45^,^46^,^50^ or nuclear genes^18^,^39^,^57^. The monophyly of Trombidiformes, however, was rejected in our analyses (Figure S1, Figure S2). The monophyly of Trombidifromes was also rejected in a recent study by Gu *et al.* based on the mt genome sequences of 16 species of Acariformes mites due to spider mites (Tetranychidae) grouped with sarcoptiform mites^50^. In our analyses, when the two species of eriophyoid mites were excluded, the rest of the Trombidiformes species were always together in a monophyletic group. Our results thus raised the need for further phylogenetics studies on Acariformes mites with more taxa included especially from Tydeoidea and Eupodoidea, which were thought to be the sister groups of Eriophyoidea^60^. Further morphological studies are also necessary to elucidate the position of eriophyoid mites in Acariformes.

In conclusion, we sequenced the mt genomes of two species of eriophyoid mites, *P. taishanensis* and *E. sabinae*. These two mites have the least rearranged mt genomes seen in the Acariformes and have highly truncated mt tRNAs. Our comparison between the eriophyoid mites and other mites and ticks showed that the most recent common ancestor of Acariformes mites retained the ancestral pattern of mt gene arrangement of arthropods with slight modifications. The truncation of three tRNAs (for cysteine, phenylalanine and histidine, respectively) likely occurred once in the common ancestor of Acariformes mites. Truncation of other tRNAs, however, occurred multiple times independently in different lineages of Acariformes mites. Our phylogenetic analyses of mt genome sequences rejected the monophyly of the order Trombidiformes when eriophyoid mites were included. Further phylogenetics studies on Acariformes mites including more taxa from different lineages is needed to clarify the position of eriophyoid mites.

## Methods

### Collection of mites

*E*. *sabinae* and *P*. *taishanensis* were collected in May 2013 in Nanjing, China. *E*. *sabinae* was collected from *Juniperus chinensis* (Cupressaceae) (China savin), while *P*. *taishanensis* from *Cedrus deodara* (Pinaceae) (Deodar cedar). Mite samples were either used immediately for DNA extraction or were preserved in 100% ethanol at −20 °C prior to DNA extraction. Samples of each eriophyoid species were also mounted to slides as voucher, using modified Berlese medium^61^ for morphological check with Zeiss A2 (microphoto camera AxioCam MRc) microscope. All of the specimens and vouchers were deposited at the Arthropod Collection, Department of Entomology, Nanjing Agricultural University, China.

### DNA extraction, mt genome amplification and sequencing

Genomic DNA was extracted from both individual and pooled specimens for each species, using a DNeasy Blood and Tissue Kit (QIAGEN), following the modified protocol^62^. For *E*. *sabinae*, a 658-bp fragment of *cox1* and a 413-bp fragment of *rrnL* were initially amplified by PCR with the primer pairs LCO1490–HCO2198^63^ and LRJ12287–LRN13398^64^ (see Additional file Table S2). PCR products were purified and sequenced directly using Sanger method at Majorbio (Shanghai, China). Specific primers for *E*. *sabinae*, ECOISR3 and E16SSR2, were designed from the sequences of the *cox1* and *rrnL* fragments, respectively. PCR with these two primers produced a 1.7-kb amplicon, which was sequenced using Sanger method at Majorbio. Another pair of primers, PF2–PR2, were designed from the sequence of the 1.7-kb amplicon. An 11.2-kb amplicon was produced with PF2–PR2 primer pair and was sequenced with Illumina Hiseq 2000 platform at the Beijing Genome Institute, Hong Kong (BGI-HK).

For *P*. *taishanensis*, a 658-bp fragment of *cox1* and a 407-bp fragment of *rrnL* were initially amplified by PCR with the primer pairs LCO1490-HCO2198^63^ and LRJ12287–LRN13398^64^ (see Additional file Table S2). The PCR products were purified and sequenced directly using Sanger method at Majorbio. Two pairs of specific primers, TYCB3R2-TY16sR1 and RTYCB3F5-RTY16sF4, were designed from the sequences of the *cox1* and *rrnL* fragments. The PCR with TYCB3R2-TY16sR1 produced a 1.7-kb amplicon, which was sequenced using Sanger method at Majorbio. The PCR with RTYCB3F5-RTY16sF4 produced an 11.2-kb amplicon, which was sequenced with Illumina Hiseq 2000 platform at the BGI-HK.

The initial PCRs contained 12.5 μL of PCR SuperMix (Transgen Biotech Co., Ltd., Beijing, China), 2 μl of template DNA, and 1.25 μM of each primer, in a total volume of 25 μL. The PCR cycling conditions were: 3-min denaturation at 96 °C; 35 cycles of 10-sec denaturation at 95 °C, 30-sec annealing at 46 °C and 1.5-min extension at 72 °C; 5-min final extension at 72 °C; and then held at 4 °C. PCR products were checked on 1% agarose gel. PrimeSTAR GXL DNA polymerase (TAKARA) was used in the long PCRs with the cycling conditions: 98 °C for 10 sec, 68 °C for 2 to 10 min (depends on the length of regions between *rrnL* and *cox1*). The reaction mixture contained 0.5 μl GXL DNA Polymerase, 5 μl buffer, 2 μl dNTP mixture, 0.75 μl of each primer, 1 μl of template DNA and Milli-Q water added to total volume of 25 μl. Positive and negative controls were executed with each PCR. PCR products were checked on 1% agarose gel. PCR products were purified with QIAquick Spin PCR Purification Kit (QIAGEN).

### Assembly of Illumina sequence-reads, gene identification and gene rearrangement analysis

Illumina sequence-reads obtained from the mt genome amplicons of *P*. *taishanensis* and *E*. *sabinae* were assembled into contigs with Geneious 6.1.6 (Biomatters Ltd.). The transfer RNA (tRNA) genes were identified using tRNAscan-SE^37^ and ARWEN^38^ or identified manually based on anticodons and secondary structures. tRNA genes of the two eriophyoid mites were verified by comparison of secondary structures and conserved nucleotide sequences with those of the Acari species reported in published literature. PCGs were identified by open reading frame search in Geneious and BLAST searches of GenBank^65^. The two rRNA genes, *rrnL* and *rrnS*, were also identified by BLAST searches of GenBank based on sequence similarity and conserved sequence motifs. The start and stop nucleotides of *rrnL* and *rrnS* cannot be determined exactly and were assumed to be immediately after their upstream genes and before their downstream genes. Breakpoints were calculated with CREx^36^ web server (http://pacosy.informatik.uni-leipzig.de/crex) as a measure of the extent of mt gene rearrangement; the two eriophyoid mites were compared with the hypothetical ancestor of arthropods (see Table 3 for details). The nucleotide sequences of mt genomes of *P*. *taishanensis* and *E*. *sabinae* have been deposited in GenBank under accession numbers KR604967 and KR604966.

### Phylogenetic analyses

Sequences of the mt genomes of 64 Acari species, including 39 Parasitiformes mites^10^,^11^,^12^,^13^,^14^,^15^,^16^,^17^,^18^,^19^,^20^,^21^,^22^,^66^,^67^ and 25 Acariformes mites^9^,^11^,^34^,^35^,^42^,^43^,^44^,^45^,^46^,^47^,^48^,^49^,^50^,^68^, and two outgroup species were retrieved from GenBank (see Additional file Table S3). We used two horseshoe crabs, *Limulus polyphemus*^69^ and *Carcinoscorpius rotundicauda*, as outgroups. Horseshoe crabs have ancestral gene arrangement of arthropod and are in the same subphylum, Cheilcerata, as mites and ticks. To investigate the phylogenetic position of Acari in Arachnida, 18 more mt genomes were retrieved from GenBank, including two species of Amblypygi^30^, four species of Araneae^26^,^30^, two species of Opiliones^30^,^70^, three species of Scorpiones^29^,^30^, two species of Solifugae^30^,^71^, one species of Thelyphonida^30^, two species of Pseudoscorpiones^28^ and two species of Ricinulei^71^. Amino acid sequences of the PCGs were aligned individually using MAFFT v7.205^72^ web server (http://mafft.cbrc.jp/alignment/server/) with G-INS-i strategy for global homology and manually inspected before concatenation. Nucleotide sequences of PCGs were aligned using TranslatorX^73^ web server (http://translatorx.co.uk/) using MAFFT to compute the protein alignments. rRNA and tRNA genes were aligned individually using Muscle algorithm implemented in MEGA 6.06^74^; large gaps and ambiguous sites were deleted manually.

We analyzed the datasets of mt genome sequences as two types of matrix: amino acid sequences of PCGs and nucleotide sequences of all genes (13 PCGs, 2 rRNA genes and 22 tRNA genes). The datasets were partitioned by genes, by codon positions and optimal partitioning as determined by PartitionFinder (Table S4). Amino acid sequences were partitioned by 13 genes. Nucleotide sequences partitioned by genes resulted in 16 datasets: 13 PCGs, 2 rRNA genes and concatenated tRNA genes. Partitioning by codon positions resulted in six datasets: one for each base of codons, two for each rRNA gene and one for the combined tRNAs. Both types of partitioned nucleotide sequences were run twice independently, once with the third codon positions of PCGs included and once without the third codon positions. To avoid redundant sampling of each genus, which may potentially affect a complex lower-level phylogeny, two representative species from each genus (*Argas*, *Ornithodoros*, *Amblyomma*, *Haemaphysalis*, *Ixodes*, *Rhipicephalus*, *Tetranychus*, *Leptotrombidium*) were included in the analyses. The reduced datasets included 43 taxa and only Baysian inference was run based on the best models found by PartitionFinder. To check if RNA genes could affect the topology, we also ran Baysian inference with the nucleotide sequences of the 13 PCGs based on the best models found by PartitionFinder. The datasets with 18 additional Arachnid mt genomes were ran in ML and Baysian analyses with nucleotide sequences of the 13 PCGs by 13 partitions.

The best models of the datasets were predicted by jModelTest 2.1.1^75^ and PartitionFinder v1.1.1^76^, using the Bayesian Information Criterion (BIC). PartitionFinder was set using unlinked branch lengths, greedy search for nucleotide sequences and amino acid sequences. MtRev + I + G + F was chosen as the best amino acid substitution model for most PCGs, except for *nad4*, for which JTT + I + G + F was chosen as the best model. Nucleotide substitution model GTR + I + G was chosen as the best of 16 partitions. The ML analyses were performed using the GTRGAMMA model for nucleotide partitions and MtRev + I + G + F (JTT + I + G + F for *nad4*) for amino acid partitions in the program raxmlGUI1.3^77^,^78^. For nodal support evaluation, a nonparametric bootstrap with 1,000 replicates was used. Mixed-model Bayesian analyses were performed with MrBayes 3.2.2^79^ using separate data partitions. For BI, one cold chain and three heated chains were run with the combined dataset for 2 million generations. The average standard deviation of split frequencies fell down quickly. After 0.2 million generations, the average standard deviation number was below 0.01 in most of the BI trees. The convergence of the parameter estimates was performed with Tracer v1.6. The consensus three was edited with FigTree1.4.0.

## Additional Information

**How to cite this article**: Xue, X.-F. *et al.* Mitochondrial genome evolution and tRNA truncation in Acariformes mites: new evidence from eriophyoid mites. *Sci. Rep.* **6**, 18920; doi: 10.1038/srep18920 (2016).

## Supplementary Material

Supplementary Information

## Figures and Tables

**Table 1:** Mitochondrial genome organization of *Phyllocoptes taishanensis* (Pt) and *Epitrimerus sabinae* (Es).

Gene	Strand	Position and intergenic nucleotides^a^		Size		Start codon		Stop codon		Anti codon
		Pt	Es	Pt	Es	Pt	Es	Pt	Es	
cox1	J	1–1554 (10)	1–1533 (14)	1554	1533	CTG	ATT	TAA	TAA	
cox2	J	1555–2220 (0)	1533–2198 (−1)	666	666	ATG	ATG	TAG	TAA	
trnK	J	2221–2280 (0)	2411–2471 (−10)	60	61					TTT
trnD	J	2279–2332 (−2)	2201–2254 (2)	54	54					GTC
atp8	J	2333–2485 (0)	2256–2420 (1)	153	165	CTG	ATT	TAA	TAA	
atp6	J	2485–3129 (−1)	2566–3207 (0)	645	642	ATG	ATG	TAA	TAA	
cox3	J	3129–3914 (−1)	3207–3998 (−1)	786	792	ATG	ATG	TAA	TAG	
trnG	J	3909–3966 (−6)	3987–4044 (−12)	58	58					TCC
nad3	J	3966–4299 (−1)	4043–4381 (−2)	334	339	ATC	ATG	T	TAA	
trnA	J	4300–4346 (0)	4381–4433 (−1)	47	53					TGC
trnR	J	4343–4393 (−4)	4425–4476 (−9)	51	52					TCG
trnN	J	4383–4436 (−11)	4466–4518 (−11)	54	53					GTT
trnS1	J	4430–4483 (−7)	4514–4568 (−5)	54	55					TCT
trnI	J	4479–4528 (−5)	4564–4612 (−5)	50	49					GAT
trnE	J	4529–4582 (0)	4613–4665 (0)	54	53					TTC
trnF	N	4581–4632 (−2)	4663–4716 (−3)	52	54					GAA
nad5	N	4633–6222 (0)	4716–6303 (−1)	1590	1588	ATT	ATT	TAA	T	
trnH	N	6235–6289 (12)	6313–6366 (9)	55	54					GTG
nad4	N	6291–7526 (1)	6383–7621 (16)	1236	1239	ATG	ATG	TAA	TAA	
nad4L	N	7526–7789 (−1)	7621–7878 (−1)	264	258	ATT	ATC	TAA	TAA	
trnP	N	7799–7852 (9)	7888–7941 (9)	54	54					TGG
nad6	J	7852–8287 (−1)	7942–8385 (0)	436	444	ATG	ATG	T	TAA	
trnT	J	8287–8341 (−1)	8366–8421 (−20)	55	56					TGT
cob	J	8346–9437 (4)	8423–9515 (1)	1092	1093	ATC	ATA	TAA	T	
trnS2	J	9437–9484 (−1)	9516–9565 (0)	48	50					TGA
nad1	N	9486–10370 (1)	9566–10442 (0)	885	877	ATA	ATA	TAG	T	
trnL2	N	10371–10429 (0)	10449–10511 (6)	59	63					TAA
trnL1	N	10431–10489 (1)	10509–10567 (−3)	59	59					TAG
trnY	J	10490–10536 (0)	10570–10616 (2)	47	47					GTA
trnQ	J	10539–10591 (2)	10624–10676 (7)	53	53					TTG
rrnS	J	10592–11255 (0)	10677–11342 (0)	664	666					
trnV	J	11256–11306 (0)	11343–11396 (0)	51	54					TAC
rrnL	J	11307–12310 (0)	11397–12401 (0)	1004	1005					
CR		12311–12357 (0)	2472–2565 (0)	47	94					
trnW	J	12358–12412 (0)	12402–12468 (0)	55	67					TCA
nad2	J	12413–13352 (0)	12470–13400 (1)	940	931	ATT	ATA	T	T	
trnM	J	13353–13413 (0)	13401–13462 (0)	61	62					CAT
trnC	N	13413–13465 (−1)	13464–13517 (1)	53	54					GCA

^a^Negative numbers indicate overlapping nucleotides between adjacent genes.

**Table 2:** Mitochondrial gene-arrangement breakpoints relative to the hypothetical ancestor of arthropods.

Superorder	Order	Taxon	Number of breakpoints
Parasitiformes	Ixodida	Argasidae (11 species)	0
Parasitiformes	Ixodida	Ixodidae (16 species)^a^	7
Parasitiformes	Mesostigmata	*Stylochyrus rarior*	7
Acariformes	Trombidiformes	*Phyllocoptes taishanensis*	13
Parasitiformes	Mesostigmata	*Varroa destructor*	14
Acariformes	Trombidiformes	Demodicidae (2 species)	15
Acariformes	Trombidiformes	*Epitrimerus sabinae*	16
Acariformes	Sarcoptiformes	Sarcoptiformes (6 species)	24
Acariformes	Trombidiformes	*Ascoschoengastia* sp.	25
Acariformes	Trombidiformes	*Walchia hayashii*	25
Acariformes	Trombidiformes	*Unionicola parkeri*	25
Acariformes	Trombidiformes	*Unionicola foili*	27
Acariformes	Trombidiformes	*Leptotrombidium pallidum*	28
Acariformes	Trombidiformes	*Leptotrombidium akamushi*^b^	28
Acariformes	Trombidiformes	Tetranychidae (10 species)	31
Parasitiformes	Mesostigmata	*Phytoseiulus persimilis*	32

^a^*Ixodes* species were not included, due to they have the same mt gene arrangement of Argasidae.

^b^*Leptotrombidium deliense* has the same gene arrangement as *Leptotrombidium akamushi*. All of the mt genomes included for comparison have the full set of 37 genes.

**Table 3:**
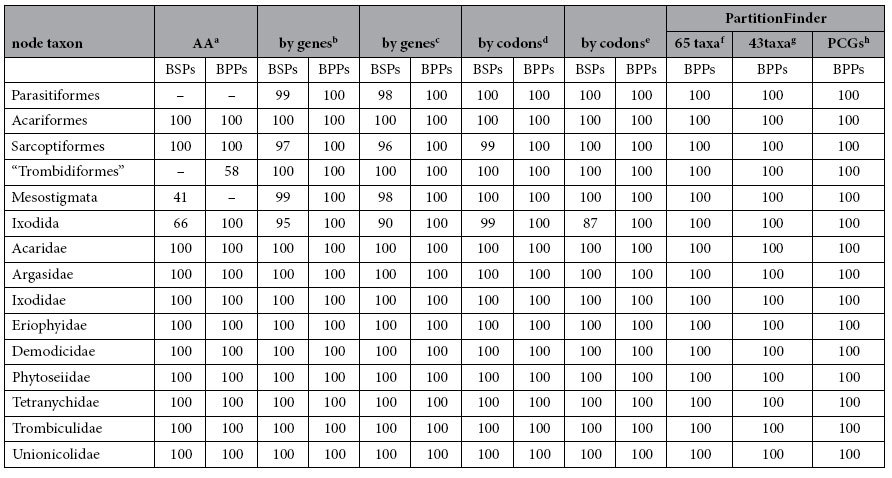
Maximum likelihood bootstrap proportions (BSPs) and Bayesian posterior probabilities (BPPs) for nodes in differently partitioned trees. Nodes are labeled as in Fig. 4. Inapplicable nodes are indicated with.

^a^Amino acids partitioned by 13 genes.

^b^nucleotide sequences by 16 partitions.

^c^nucleotide sequences by 16 partitions except for the third positions of PCGs.

^d^nucleotide sequences by 6 partitions.

^e^nucleotide sequences by 5 partitions except for the third positions of PCGs.

^f^the best partitions of nucleotide sequences found by PartitionFinder.

^g^the best partitions of nucleotide sequences found by PartitionFinder with truncated taxon

^h^the best partitions of nucleotide sequences found by PartitionFinder with 13PCGs. “Trombidiformes” does not contain species of Eriophyoidea.
