# Supplementary material for: Mitochondrial genome evolution and tRNA truncation in Acariformes mites: new evidence from eriophyoid mites
Source: Sci Rep. 2016 Jan 6;6:18920. doi: 10.1038/srep18920 (PMC4702108; doi:10.1038/srep18920)
Supplement: Supplementary Information [file srep18920-s1.pdf]

Mitochondrial genome evolution and tRNA truncation in Acariformes mites: new evidence from eriophyoid mites

Xiao-Feng Xue[1,2], Jing-Feng Guo[1], Yan Dong[1], Xiao-Yue Hong[1] & Renfu Shao[2]

[1]Department of Entomology, Nanjing Agricultural University, Nanjing, Jiangsu 210095, China, [2]GeneCology Research Centre, Faculty of Science, Health, Education and Engineering, University of the Sunshine Coast, Maroochydore, Queensland 4556, Australia.

Correspondence and requests for materials should be addressed to X.-F. X. (xfxue@njau.edu.cn) and R.S. (rshao@usc.edu.au)

Corresponding author: Tel.: +86 25 84395868

Supplementary Figure S1. Maximum likelihood credibility trees inferred with: A) nucleotide sequences by 16 partitions; B) nucleotide sequences by 6 partitions; C) nucleotide sequences by 16 partitionsexcluding the third codon positions of PCGs; D) nucleotide sequences by 6 partitions excluding the third positions of the PCGs; E) amino acid sequences partitioned by 13 genes; and F) nucleotide sequences of PCGs of 86 taxa by 13 partitions. Maximum likelihood bootstrap proportions (BSPs) are indicated at each node.

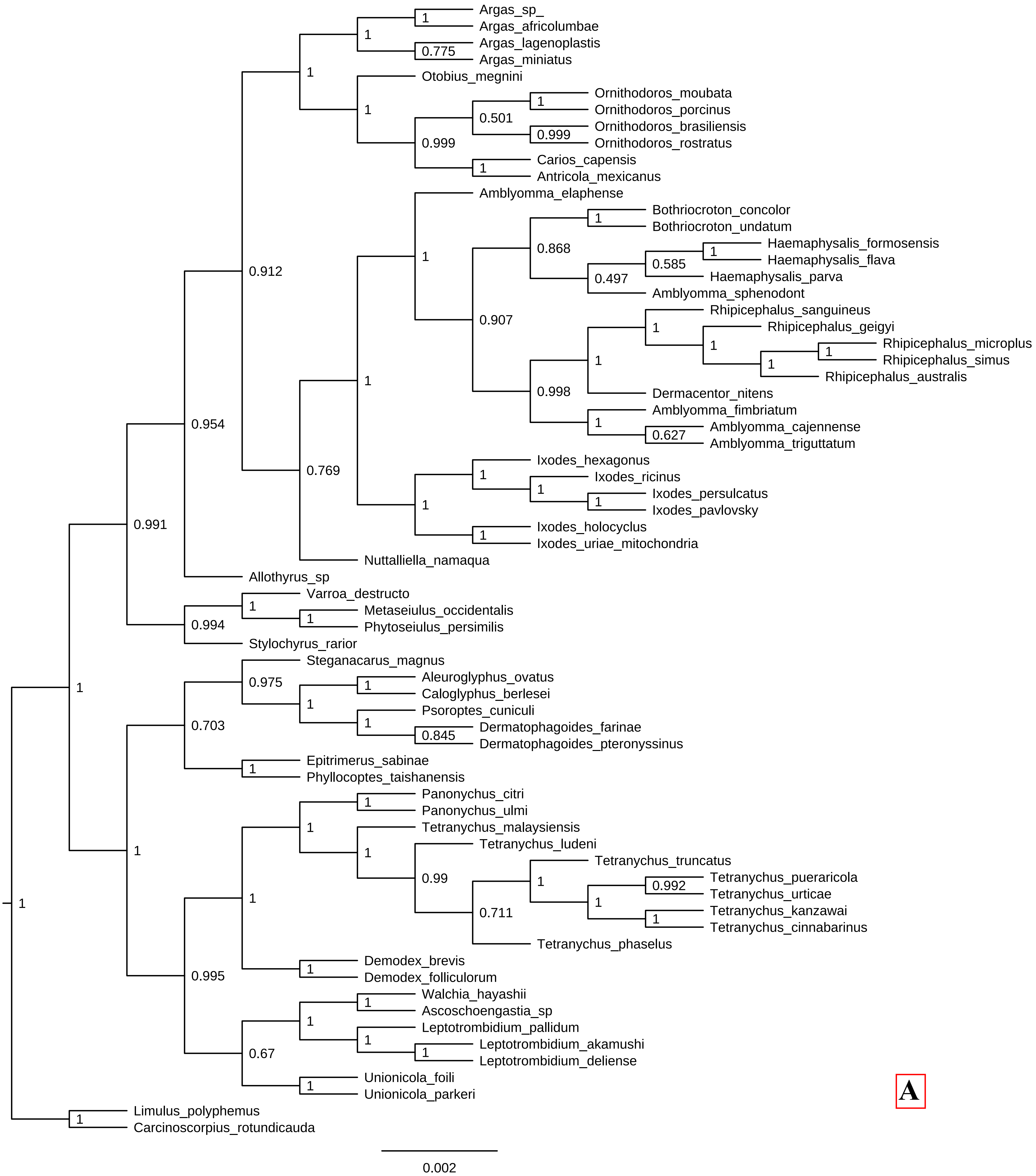
Argas_sp_
Argas_africolumbae
Argas_lagenoplastis
Argas_miniatus
Otobius_megnini
Ornithodoros_moubata
Ornithodoros_porcinus
Ornithodoros_brasiliensis
Ornithodoros_rostratus
Carios_capensis
Antricola_mexicanus
Amblyomma_elaphense
Bothriocroton_concolor
Bothriocroton_undatum
Haemaphysalis_formosensis
Haemaphysalis_flava
Haemaphysalis_parva
Amblyomma_sphenodont
Rhipicephalus_sanguineus
Rhipicephalus_geigyi
Rhipicephalus_microplus
Rhipicephalus_simus
Rhipicephalus_australis
Dermacentor_nitens
Amblyomma_fimbriatum
Amblyomma_cajennense
Amblyomma_triguttatum
Ixodes_hexagonus
Ixodes_ricinus
Ixodes_persulcatus
Ixodes_pavlovsky
Ixodes_holocyclus
Ixodes_uriae_mitochondria
Nuttalliella_namaqua
Allothyrus_sp
Varroa_destructo
Metaseiulus_occidentalis
Phytoseiulus_persimilis
Stylochyrus_rarior
Steganacarus_magnus
Aleuroglyphus_ovatus
Caloglyphus_berlesei
Psoroptes_cuniculi
Dermatophagoides_farinae
Dermatophagoides_pteronyssinus
Epitrimerus_sabinae
Phyllocoptes_taishanensis
Panonychus_citri
Panonychus_ulmi
Tetranychus_malaysiensis
Tetranychus_ludeni
Tetranychus_truncatus
Tetranychus_pueraricola
Tetranychus_urticae
Tetranychus_kanzawai
Tetranychus_cinnabarinus
Tetranychus_phaselus
Demodex_brevis
Demodex_folliculorum
Walchia_hayashii
Ascoschoengastia_sp
Leptotrombidium_pallidum
Leptotrombidium_akamushi
Leptotrombidium_deliense
Unionicola_foili
Unionicola_parkeri
Limulus_polyphemus
Carcinoscorpius_rotundicauda
0.002
A

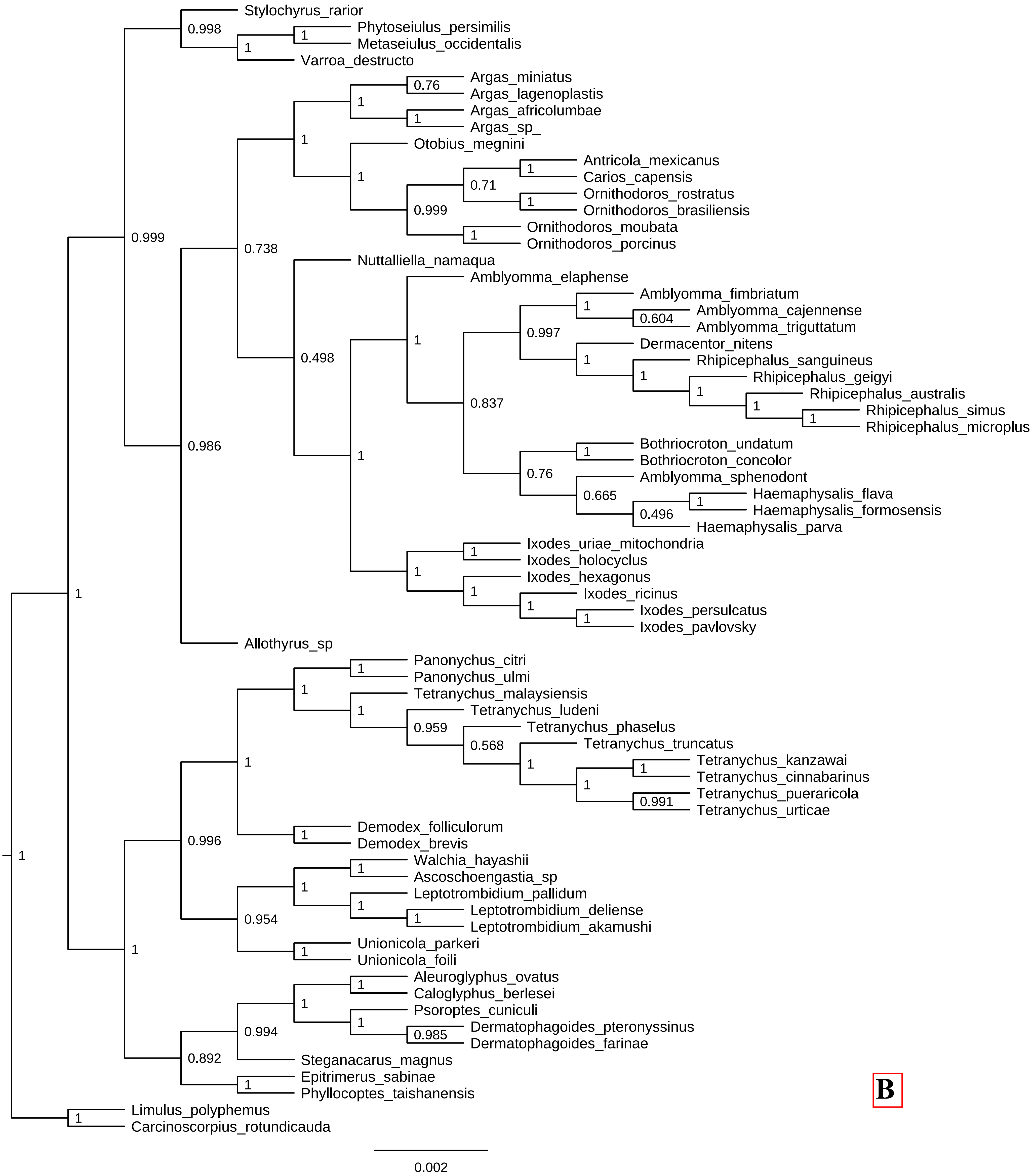

Stylochyrus_rarior
Phytoseiulus_persimilis
Metaseiulus_occidentalis
Varroa_destructo
Argas_miniatus
Argas_lagenoplastis
Argas_africolumbae
Argas_sp_
Otobius_megnini
Antricola_mexicanus
Carios_capensis
Ornithodoros_rostratus
Ornithodoros_brasiliensis
Ornithodoros_moubata
Ornithodoros_porcinus
Nuttalliella_namaqua
Amblyomma_elaphense
Amblyomma_fimbriatum
Amblyomma_cajennense
Amblyomma_triguttatum
Dermacentor_nitens
Rhipicephalus_sanguineus
Rhipicephalus_geigyi
Rhipicephalus_australis
Rhipicephalus_simus
Rhipicephalus_microplus
Bothriocroton_undatum
Bothriocroton_concolor
Amblyomma_sphenodont
Haemaphysalis_flava
Haemaphysalis_formosensis
Haemaphysalis_parva
Ixodes_uriae_mitochondria
Ixodes_holocyclus
Ixodes_hexagonus
Ixodes_ricinus
Ixodes_persulcatus
Ixodes_pavlovsky
Allothyrus_sp
Panonychus_citri
Panonychus_ulmi
Tetranychus_malaysiensis
Tetranychus_ludeni
Tetranychus_phaselus
Tetranychus_truncatus
Tetranychus_kanzawai
Tetranychus_cinnabarinus
Tetranychus_pueraricola
Tetranychus_urticae
Demodex_folliculorum
Demodex_brevis
Walchia_hayashii
Ascoschoengastia_sp
Leptotrombidium_pallidum
Leptotrombidium_deliense
Leptotrombidium_akamushi
Unionicola_parkeri
Unionicola_foili
Aleuroglyphus_ovatus
Caloglyphus_berlesei
Psoroptes_cuniculi
Dermatophagoides_pteronyssinus
Dermatophagoides_farinae
Steganacarus_magnus
Epitrimerus_sabinae
Phyllocoptes_taishanensis
Limulus_polyphemus
Carcinoscorpius_rotundicauda
0.998
0.999
0.76
0.738
0.71
0.999
0.498
0.604
0.997
0.837
0.986
0.76
0.665
0.496
0.959
0.568
0.991
0.996
0.954
0.994
0.985
0.892
1
0.002
B

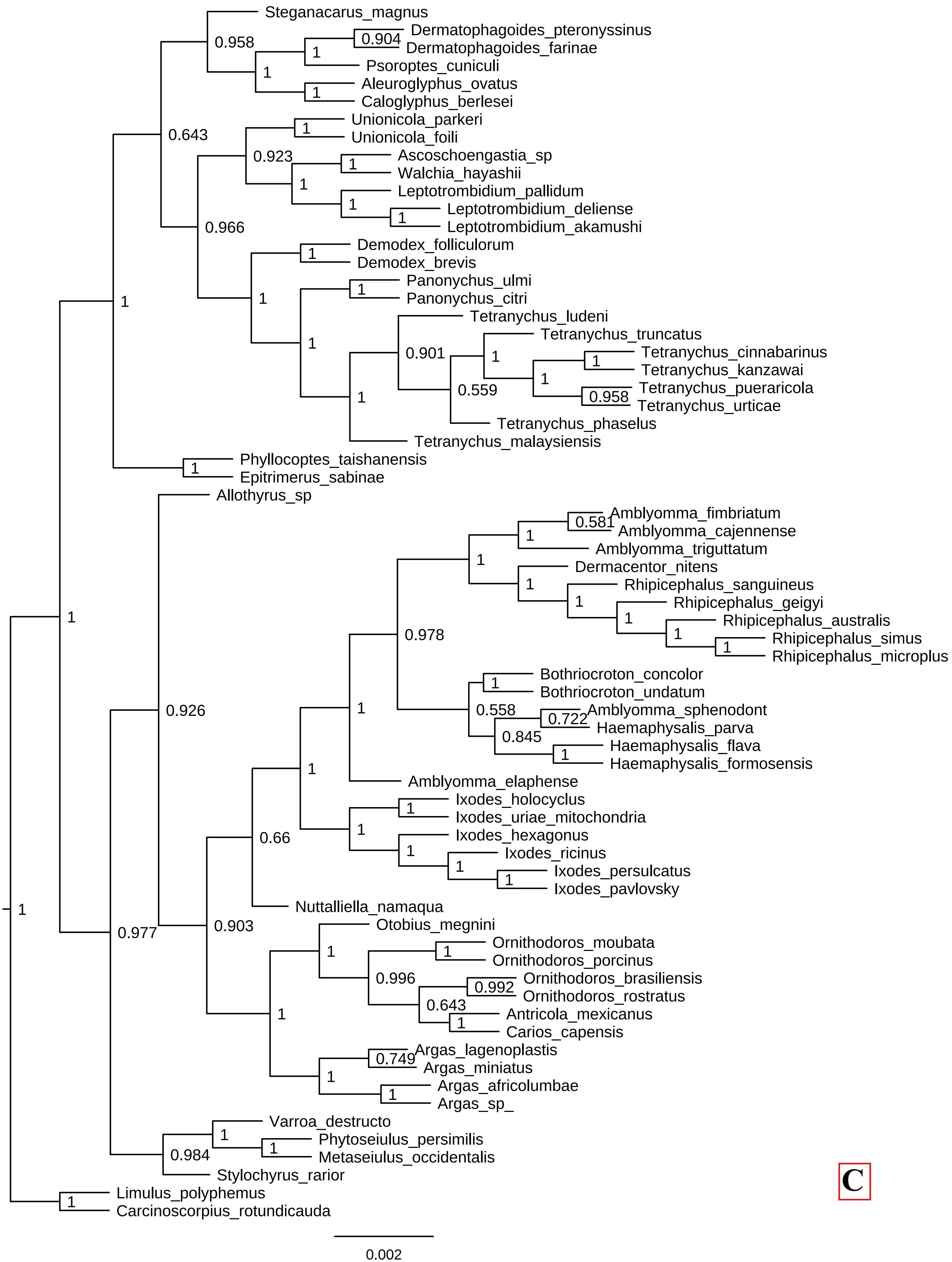
Steganacarus_magnus
Dermatophagoides_pteronyssinus
Dermatophagoides_farinae
Psoroptes_cuniculi
Aleuroglyphus_ovatus
Caloglyphus_berlesei
Unionicola_parkeri
Unionicola_foili
Ascoschoengastia_sp
Walchia_hayashii
Leptotrombidium_pallidum
Leptotrombidium_deliense
Leptotrombidium_akamushi
Demodex_folliculorum
Demodex_brevis
Panonychus_ulmi
Panonychus_citri
Tetranychus_ludeni
Tetranychus_truncatus
Tetranychus_cinnabarinus
Tetranychus_kanzawai
Tetranychus_pueraricola
Tetranychus_urticae
Tetranychus_phaselus
Tetranychus_malaysiensis
Phyllocoptes_taishanensis
Epitrimerus_sabinae
Allothyrus_sp
Amblyomma_fimbriatum
Amblyomma_cajennense
Amblyomma_triguttatum
Dermacentor_nitens
Rhipicephalus_sanguineus
Rhipicephalus_geigyi
Rhipicephalus_australis
Rhipicephalus_simus
Rhipicephalus_microplus
Bothriocroton_concolor
Bothriocroton_undatum
Amblyomma_sphenodont
Haemaphysalis_parva
Haemaphysalis_flava
Haemaphysalis_formosensis
Amblyomma_elaphense
Ixodes_holocyclus
Ixodes_uriae_mitochondria
Ixodes_hexagonus
Ixodes_ricinus
Ixodes_persulcatus
Ixodes_pavlovsky
Nuttalliella_namaqua
Otobius_megnini
Ornithodoros_moubata
Ornithodoros_porcinus
Ornithodoros_brasiliensis
Ornithodoros_rostratus
Antricola_mexicanus
Carios_capensis
Argas_lagenoplastis
Argas_miniatus
Argas_africolumbae
Argas_sp_
Varroa_destructo
Phytoseiulus_persimilis
Metaseiulus_occidentalis
Stylochyrus_rarior
Limulus_polyphemus
Carcinoscorpius_rotundicauda
0.002
C

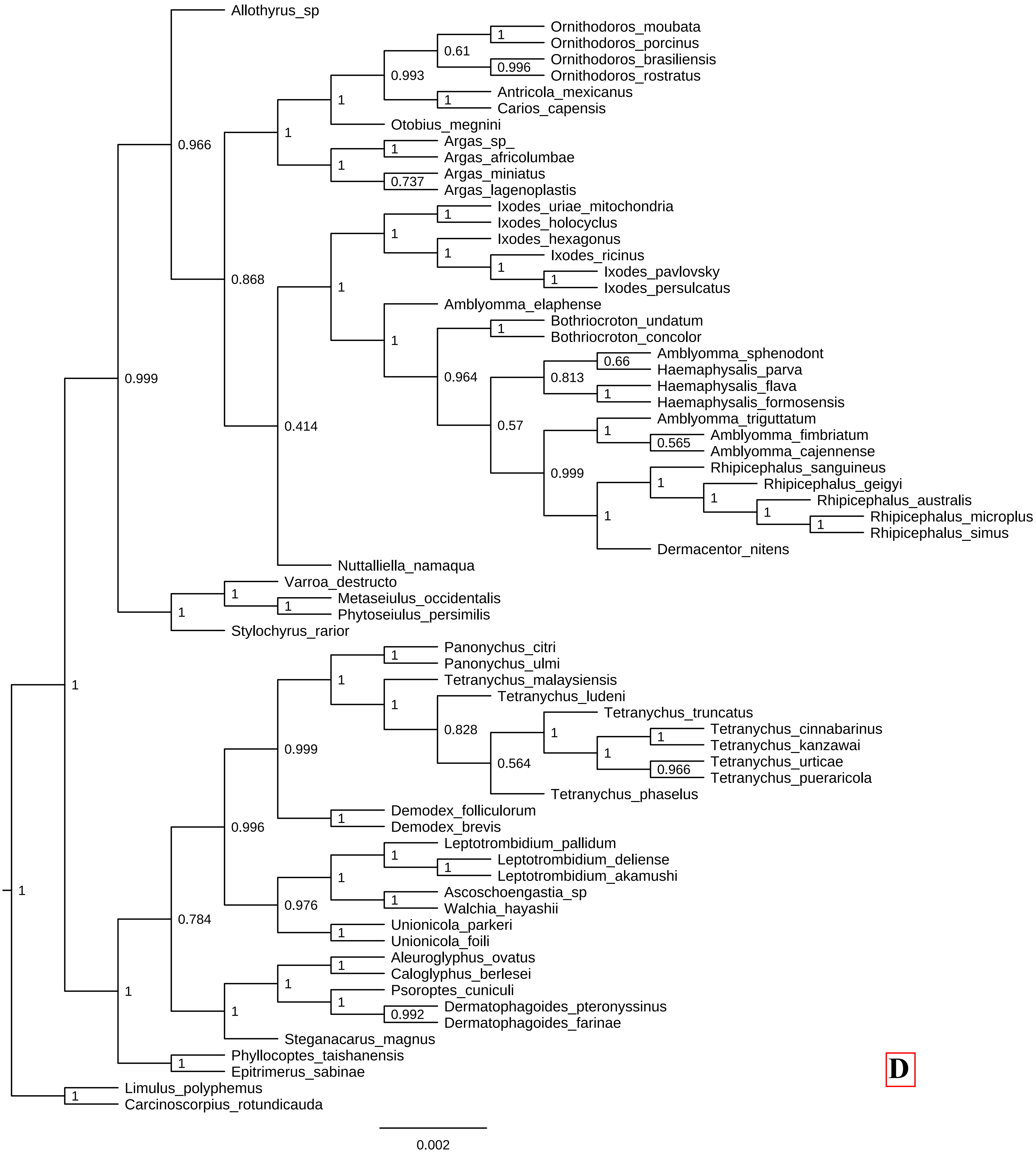
Allothyrus_sp
Ornithodoros_moubata
Ornithodoros_porcinus
Ornithodoros_brasiliensis
Ornithodoros_rostratus
Antricola_mexicanus
Carios_capensis
Otobius_megnini
Argas_sp_
Argas_africolumbae
Argas_miniatus
Argas_lagenoplastis
Ixodes_uriae_mitochondria
Ixodes_holocyclus
Ixodes_hexagonus
Ixodes_ricinus
Ixodes_pavlovsky
Ixodes_persulcatus
Amblyomma_elaphense
Bothriocroton_undatum
Bothriocroton_concolor
Amblyomma_sphenodont
Haemaphysalis_parva
Haemaphysalis_flava
Haemaphysalis_formosensis
Amblyomma_triguttatum
Amblyomma_fimbriatum
Amblyomma_cajennense
Rhipicephalus_sanguineus
Rhipicephalus_geigyi
Rhipicephalus_australis
Rhipicephalus_microplus
Rhipicephalus_simus
Dermacentor_nitens
Nuttalliella_namaqua
Varroa_destructo
Metaseiulus_occidentalis
Phytoseiulus_persimilis
Stylochyrus_rarior
Panonychus_citri
Panonychus_ulmi
Tetranychus_malaysiensis
Tetranychus_ludeni
Tetranychus_truncatus
Tetranychus_cinnabarinus
Tetranychus_kanzawai
Tetranychus_urticae
Tetranychus_pueraricola
Tetranychus_phaselus
Demodex_folliculorum
Demodex_brevis
Leptotrombidium_pallidum
Leptotrombidium_deliense
Leptotrombidium_akamushi
Ascoschoengastia_sp
Walchia_hayashii
Unionicola_parkeri
Unionicola_foili
Aleuroglyphus_ovatus
Caloglyphus_berlesei
Psoroptes_cuniculi
Dermatophagoides_pteronyssinus
Dermatophagoides_farinae
Steganacarus_magnus
Phyllocoptes_taishanensis
Epitrimerus_sabinae
Limulus_polyphemus
Carcinoscorpius_rotundicauda
0.966
0.61
0.993
0.996
0.737
0.868
0.964
0.813
0.66
0.57
0.565
0.999
0.414
0.828
0.564
0.966
0.996
0.976
0.992
0.784
0.002
D

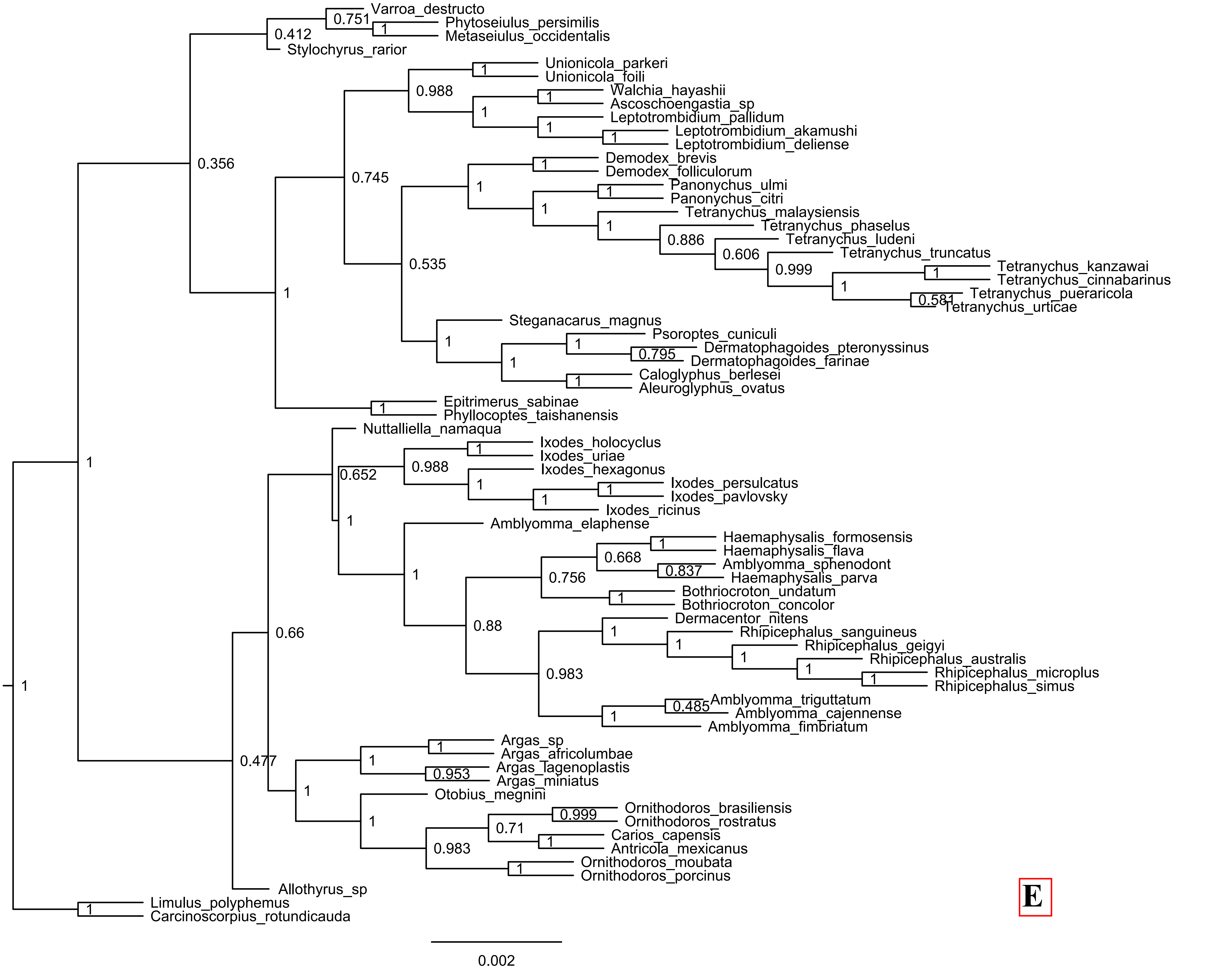
Varroa_destructo
Phytoseiulus_persimilis
Metaseiulus_occidentalis
Stylochyrus_rarior
Unionicola_parkeri
Unionicola_foili
Walchia_hayashii
Ascoschoengastia_sp
Leptotrombidium_pallidum
Leptotrombidium_akamushi
Leptotrombidium_deliense
Demodex_brevis
Demodex_folliculorum
Panonychus_ulmi
Panonychus_citri
Tetranychus_malaysiensis
Tetranychus_phaselus
Tetranychus_ludeni
Tetranychus_truncatus
Tetranychus_kanzawai
Tetranychus_cinnabarinus
Tetranychus_pueraricola
Tetranychus_urticae
Steganacarus_magnus
Psoroptes_cuniculi
Dermatophagoides_pteronyssinus
Dermatophagoides_farinae
Caloglyphus_berlesei
Aleuroglyphus_ovatus
Epitrimerus_sabinae
Phyllocoptes_taishanensis
Nuttalliella_namaqua
Ixodes_holocyclus
Ixodes_uriae
Ixodes_hexagonus
Ixodes_persulcatus
Ixodes_pavlovsky
Ixodes_ricinus
Amblyomma_elaphense
Haemaphysalis_formosensis
Haemaphysalis_flava
Amblyomma_sphenodont
Haemaphysalis_parva
Bothriocroton_undatum
Bothriocroton_concolor
Dermacentor_nitens
Rhipicephalus_sanguineus
Rhipicephalus_geigyi
Rhipicephalus_australis
Rhipicephalus_microplus
Rhipicephalus_simus
Amblyomma_triguttatum
Amblyomma_cajennense
Amblyomma_fimbriatum
Argas_sp
Argas_africolumbae
Argas_lagenoplastis
Argas_miniatus
Otobius_megnini
Ornithodoros_brasiliensis
Ornithodoros_rostratus
Carios_capensis
Antricola_mexicanus
Ornithodoros_moubata
Ornithodoros_porcinus
Allothyrus_sp
Limulus_polyphemus
Carcinoscorpius_rotundicauda
0.002
E

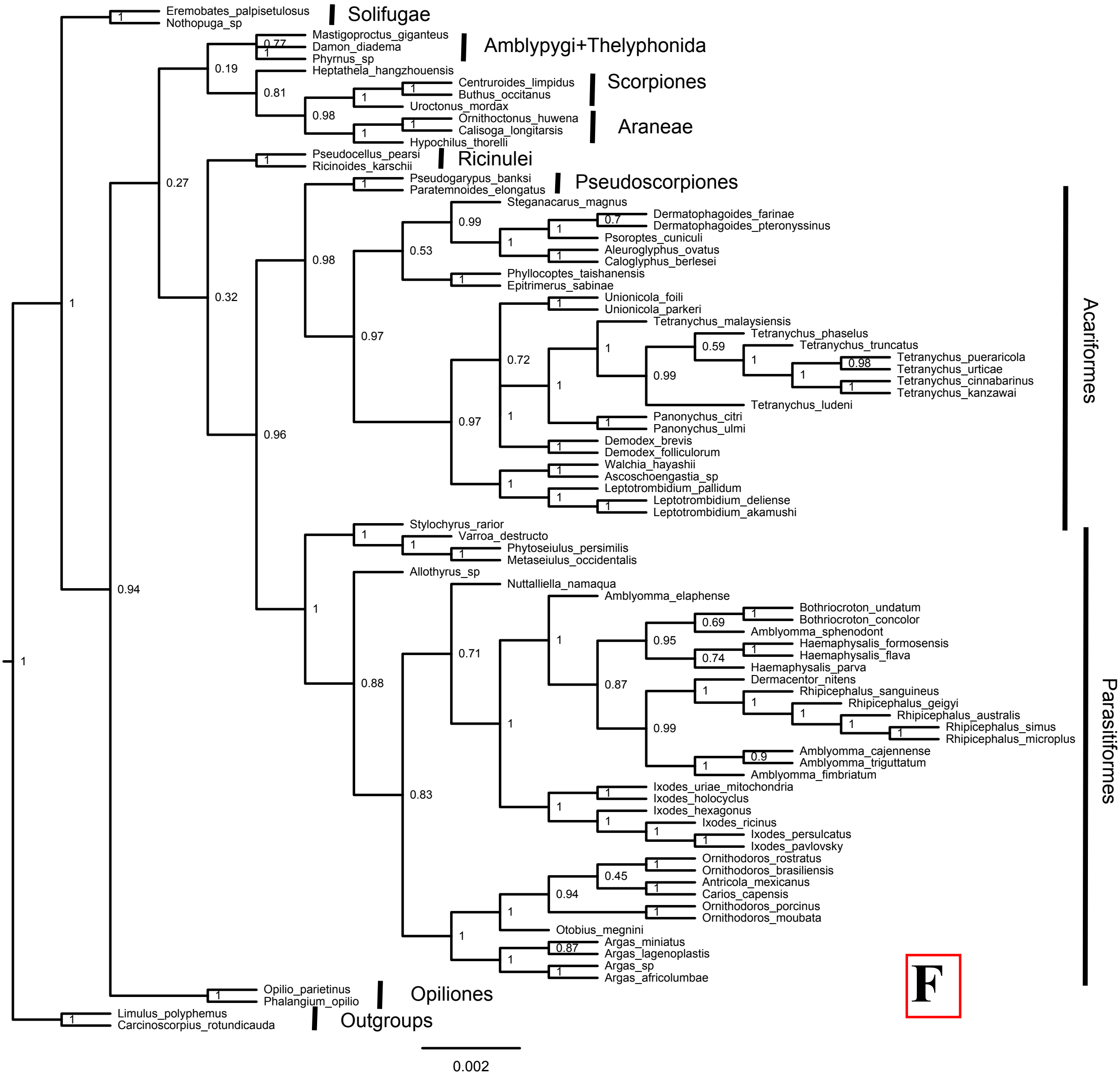
Solifugae
Amblypygi+Thelyphonida
Scorpiones
Araneae
Ricinulei
Pseudoscorpiones
Acariformes
Parasitiformes
Opiliones
Outgroups
Eremobates_palpisetulosus
Nothopuga_sp
Mastigoproctus_giganteus
Damon_diadema
Phyrnus_sp
Heptathela_hangzhouensis
Centruroides_limpidus
Buthus_occitanus
Uroctonus_mordax
Ornithoctonus_huwena
Calisoga_longitarsis
Hypochilus_thorelli
Pseudocellus_pearsi
Ricinoides_karschii
Pseudogarypus_banksi
Paratemnoides_elongatus
Steganacarus_magnus
Dermatophagoides_farinae
Dermatophagoides_pteronyssinus
Psoroptes_cuniculi
Aleuroglyphus_ovatus
Caloglyphus_berlesei
Phyllocoptes_taishanensis
Epitrimerus_sabinae
Unionicola_foili
Unionicola_parkeri
Tetranychus_malaysiensis
Tetranychus_phaselus
Tetranychus_truncatus
Tetranychus_pueraricola
Tetranychus_urticae
Tetranychus_cinnabarinus
Tetranychus_kanzawai
Tetranychus_ludeni
Panonychus_citri
Panonychus_ulmi
Demodex_brevis
Demodex_folliculorum
Walchia_hayashii
Ascoschoengastia_sp
Leptotrombidium_pallidum
Leptotrombidium_deliense
Leptotrombidium_akamushi
Stylochyrus_rarior
Varroa_destructo
Phytoseiulus_persimilis
Metaseiulus_occidentalis
Allothyrus_sp
Nuttalliella_namaqua
Amblyomma_elaphense
Bothriocroton_undatum
Bothriocroton_concolor
Amblyomma_sphenodont
Haemaphysalis_formosensis
Haemaphysalis_flava
Haemaphysalis_parva
Dermacentor_nitens
Rhipicephalus_sanguineus
Rhipicephalus_geigyi
Rhipicephalus_australis
Rhipicephalus_simus
Rhipicephalus_microplus
Amblyomma_cajennense
Amblyomma_triguttatum
Amblyomma_fimbriatum
Ixodes_uriae_mitochondria
Ixodes_holocyclus
Ixodes_hexagonus
Ixodes_ricinus
Ixodes_persulcatus
Ixodes_pavlovsky
Ornithodoros_rostratus
Ornithodoros_brasiliensis
Antricola_mexicanus
Carios_capensis
Ornithodoros_porcinus
Ornithodoros_moubata
Otobius_megnini
Argas_miniatus
Argas_lagenoplastis
Argas_sp
Argas_africolumbae
Opilio_parietinus
Phalangium_opilio
Limulus_polyphemus
Carcinoscorpius_rotundicauda
0.002
F

Supplementary Figure S2. Bayesian maximum credibility trees inferred by: A) nucleotide sequences by 16 partitions; B) amino acid sequences partitioned by 13 genes; C) nucleotide sequences by 16 partitions, except the third positions of the PCGs; D) nucleotide sequences by 6 partitions; E) nucleotide sequences by 6 partitions, except the third positions of the PCGs; F) the best partitions of nucleotide sequences found by PartitionFinder; G) the best partitions of nucleotide sequences found by PartitionFinder with truncated taxon; H) the best partitions of nucleotide sequences found by PartitionFinder with 13PCGs; and I) nucleotide sequences of PCGs of 86 taxa by 13 partitions. Bayesian posterior probabilities (BPPs) are indicated at each node.

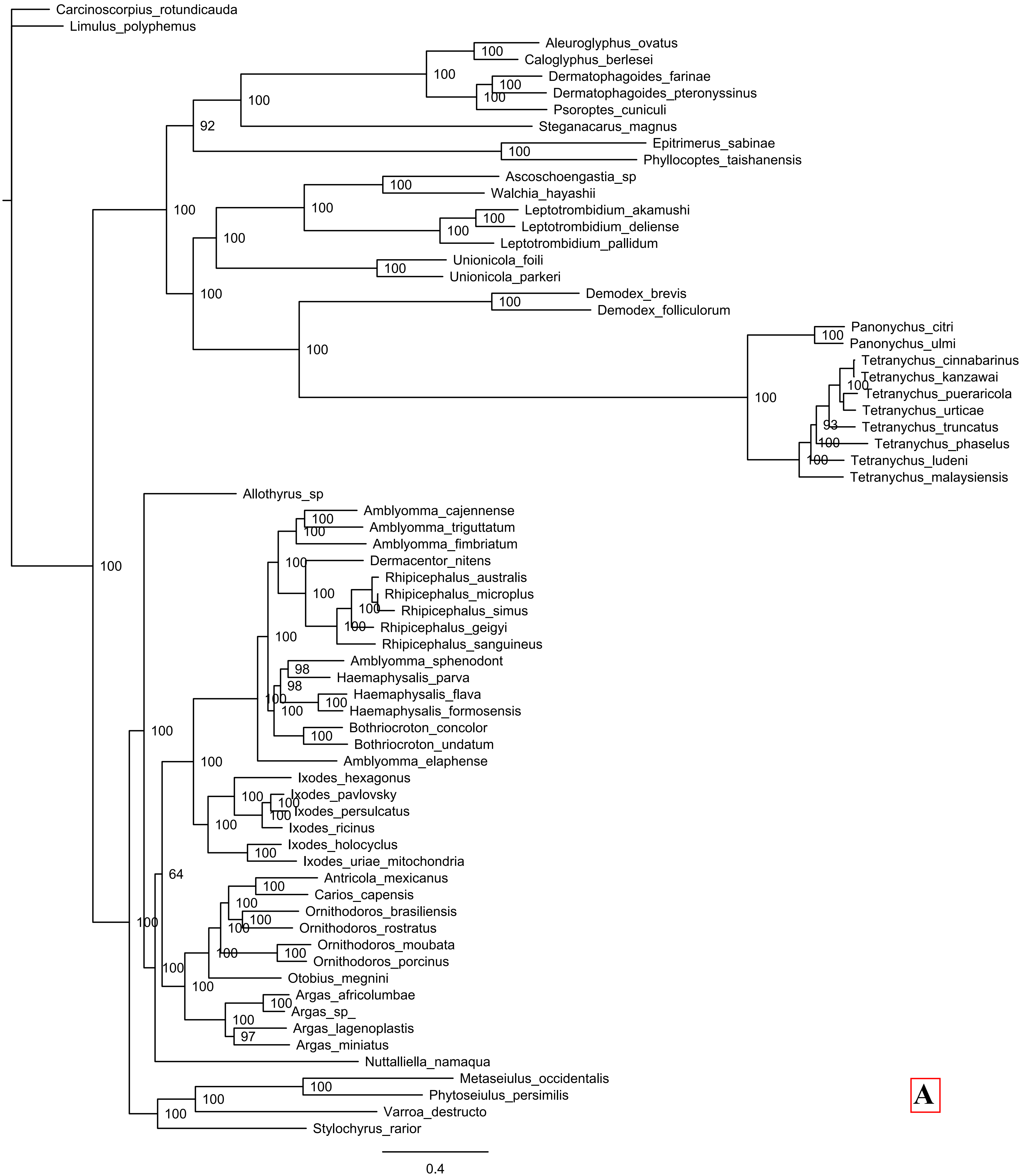

Carcinoscorpius_rotundicauda
Limulus_polyphemus
Aleuroglyphus_ovatus
Caloglyphus_berlesei
Dermatophagoides_farinae
Dermatophagoides_pteronyssinus
Psoroptes_cuniculi
Steganacarus_magnus
Epitrimerus_sabinae
Phyllocoptes_taishanensis
Ascoschoengastia_sp
Walchia_hayashii
Leptotrombidium_akamushi
Leptotrombidium_deliense
Leptotrombidium_pallidum
Unionicola_foili
Unionicola_parkeri
Demodex_brevis
Demodex_folliculorum
Panonychus_citri
Panonychus_ulmi
Tetranychus_cinnabarinus
Tetranychus_kanzawai
Tetranychus_pueraricola
Tetranychus_urticae
Tetranychus_truncatus
Tetranychus_phaselus
Tetranychus_ludeni
Tetranychus_malaysiensis
Allothyrus_sp
Amblyomma_cajennense
Amblyomma_triguttatum
Amblyomma_fimbriatum
Dermacentor_nitens
Rhipicephalus_australis
Rhipicephalus_microplus
Rhipicephalus_simus
Rhipicephalus_geigyi
Rhipicephalus_sanguineus
Amblyomma_sphenodont
Haemaphysalis_parva
Haemaphysalis_flava
Haemaphysalis_formosensis
Bothriocroton_concolor
Bothriocroton_undatum
Amblyomma_elaphense
Ixodes_hexagonus
Ixodes_pavlovsky
Ixodes_persulcatus
Ixodes_ricinus
Ixodes_holocyclus
Ixodes_uriae_mitochondria
Antricola_mexicanus
Carios_capensis
Ornithodoros_brasiliensis
Ornithodoros_rostratus
Ornithodoros_moubata
Ornithodoros_porcinus
Otobius_megnini
Argas_africolumbae
Argas_sp_
Argas_lagenoplastis
Argas_miniatus
Nuttalliella_namaqua
Metaseiulus_occidentalis
Phytoseiulus_persimilis
Varroa_destructo
Stylochyrus_rarior
0.4
A

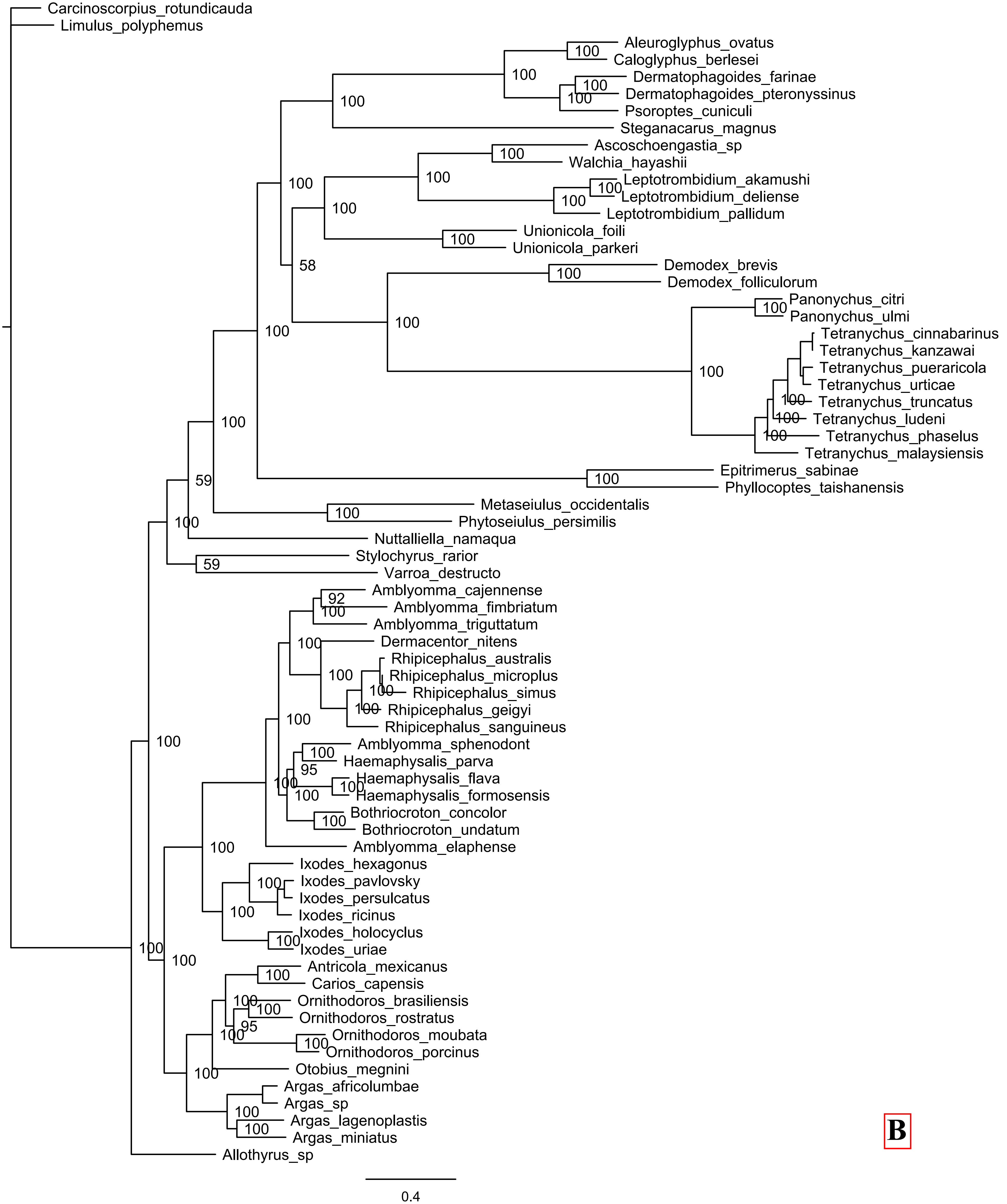
Carcinoscorpius_rotundicauda
Limulus_polyphemus
Aleuroglyphus_ovatus
Caloglyphus_berlesei
Dermatophagoides_farinae
Dermatophagoides_pteronyssinus
Psoroptes_cuniculi
Steganacarus_magnus
Ascoschoengastia_sp
Walchia_hayashii
Leptotrombidium_akamushi
Leptotrombidium_deliense
Leptotrombidium_pallidum
Unionicola_foili
Unionicola_parkeri
Demodex_brevis
Demodex_folliculorum
Panonychus_citri
Panonychus_ulmi
Tetranychus_cinnabarinus
Tetranychus_kanzawai
Tetranychus_pueraricola
Tetranychus_urticae
Tetranychus_truncatus
Tetranychus_ludeni
Tetranychus_phaselus
Tetranychus_malaysiensis
Epitrimerus_sabinae
Phyllocoptes_taishanensis
Metaseiulus_occidentalis
Phytoseiulus_persimilis
Nuttalliella_namaqua
Stylochyrus_rarior
Varroa_destructo
Amblyomma_cajennense
Amblyomma_fimbriatum
Amblyomma_triguttatum
Dermacentor_nitens
Rhipicephalus_australis
Rhipicephalus_microplus
Rhipicephalus_simus
Rhipicephalus_geigyi
Rhipicephalus_sanguineus
Amblyomma_sphenodont
Haemaphysalis_parva
Haemaphysalis_flava
Haemaphysalis_formosensis
Bothriocroton_concolor
Bothriocroton_undatum
Amblyomma_elaphense
Ixodes_hexagonus
Ixodes_pavlovsky
Ixodes_persulcatus
Ixodes_ricinus
Ixodes_holocyclus
Ixodes_uriae
Antricola_mexicanus
Carios_capensis
Ornithodoros_brasiliensis
Ornithodoros_rostratus
Ornithodoros_moubata
Ornithodoros_porcinus
Otobius_megnini
Argas_africolumbae
Argas_sp
Argas_lagenoplastis
Argas_miniatus
Allothyrus_sp
100
58
59
92
95
0.4
B

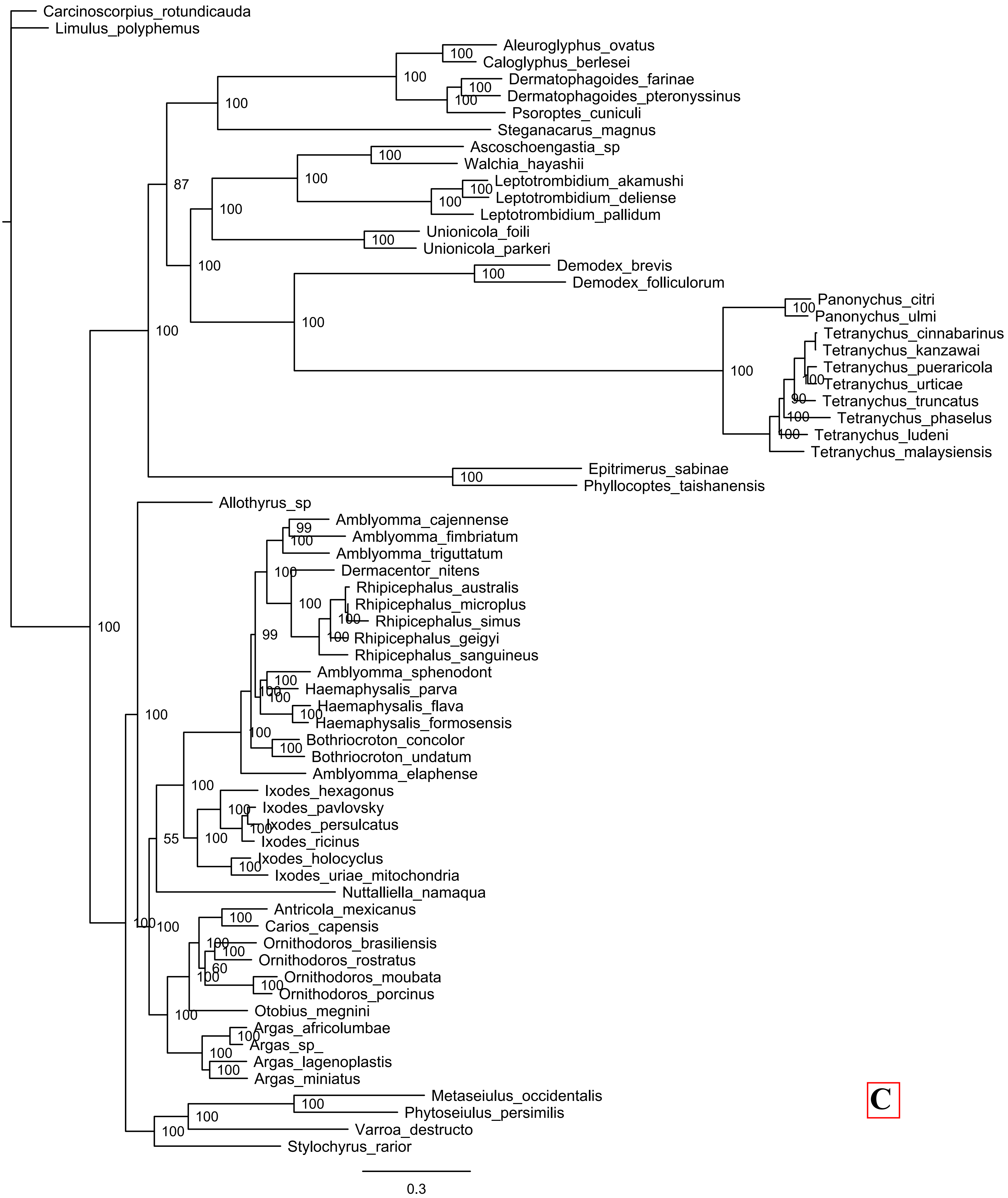
Carcinoscorpius_rotundicauda
Limulus_polyphemus
Aleuroglyphus_ovatus
Caloglyphus_berlesei
Dermatophagoides_farinae
Dermatophagoides_pteronyssinus
Psoroptes_cuniculi
Steganacarus_magnus
Ascoschoengastia_sp
Walchia_hayashii
Leptotrombidium_akamushi
Leptotrombidium_deliense
Leptotrombidium_pallidum
Unionicola_foili
Unionicola_parkeri
Demodex_brevis
Demodex_folliculorum
Panonychus_citri
Panonychus_ulmi
Tetranychus_cinnabarinus
Tetranychus_kanzawai
Tetranychus_pueraricola
Tetranychus_urticae
Tetranychus_truncatus
Tetranychus_phaselus
Tetranychus_ludeni
Tetranychus_malaysiensis
Epitrimerus_sabinae
Phyllocoptes_taishanensis
Allothyrus_sp
Amblyomma_cajennense
Amblyomma_fimbriatum
Amblyomma_triguttatum
Dermacentor_nitens
Rhipicephalus_australis
Rhipicephalus_microplus
Rhipicephalus_simus
Rhipicephalus_geigyi
Rhipicephalus_sanguineus
Amblyomma_sphenodont
Haemaphysalis_parva
Haemaphysalis_flava
Haemaphysalis_formosensis
Bothriocroton_concolor
Bothriocroton_undatum
Amblyomma_elaphense
Ixodes_hexagonus
Ixodes_pavlovsky
Ixodes_persulcatus
Ixodes_ricinus
Ixodes_holocyclus
Ixodes_uriae_mitochondria
Nuttalliella_namaqua
Antricola_mexicanus
Carios_capensis
Ornithodoros_brasiliensis
Ornithodoros_rostratus
Ornithodoros_moubata
Ornithodoros_porcinus
Otobius_megnini
Argas_africolumbae
Argas_sp_
Argas_lagenoplastis
Argas_miniatus
Metaseiulus_occidentalis
Phytoseiulus_persimilis
Varroa_destructo
Stylochyrus_rarior
C
0.3

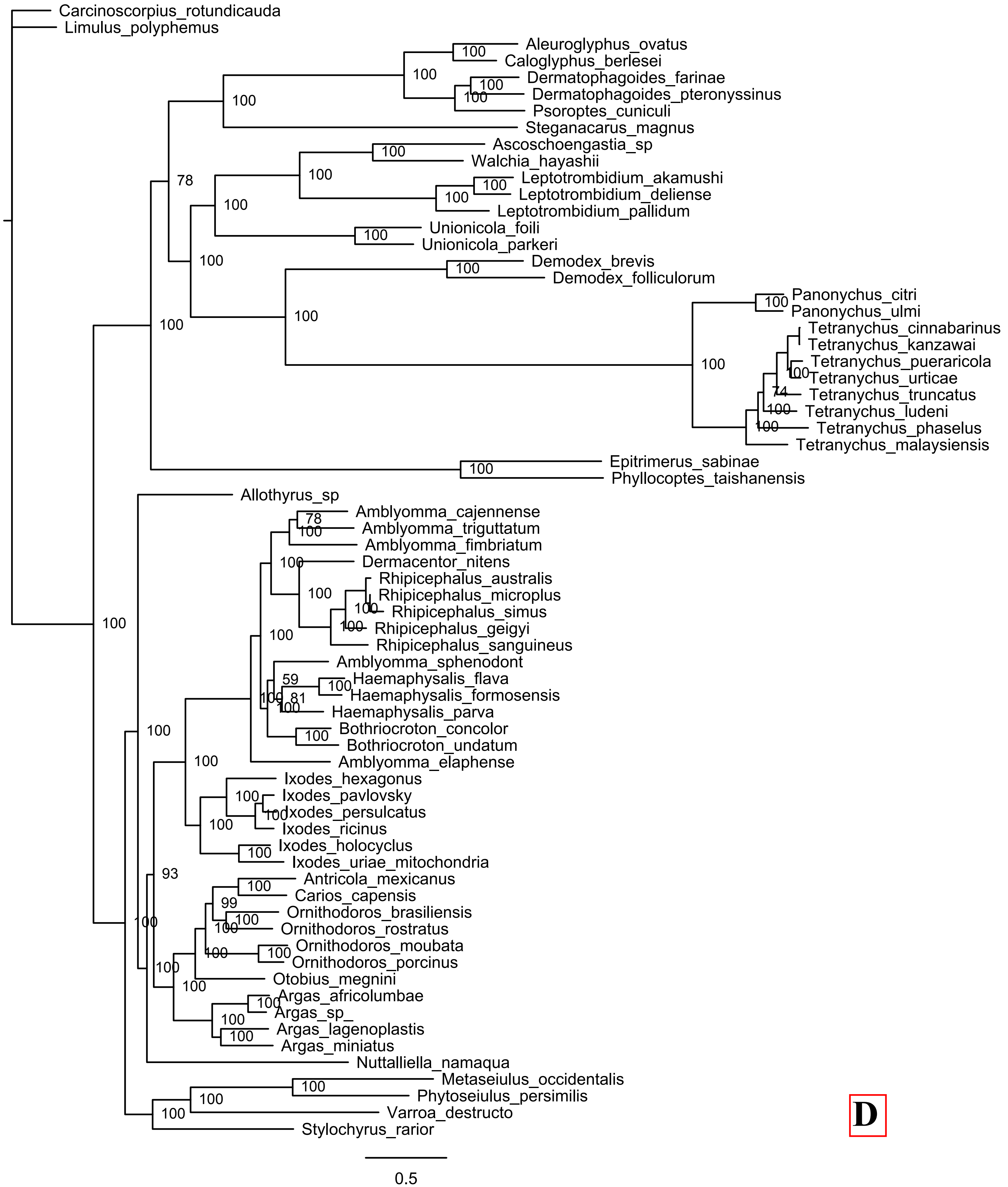

Carcinoscorpius_rotundicauda
Limulus_polyphemus
Aleuroglyphus_ovatus
Caloglyphus_berlesei
Dermatophagoides_farinae
Dermatophagoides_pteronyssinus
Psoroptes_cuniculi
Steganacarus_magnus
Ascoschoengastia_sp
Walchia_hayashii
Leptotrombidium_akamushi
Leptotrombidium_deliense
Leptotrombidium_pallidum
Unionicola_foili
Unionicola_parkeri
Demodex_brevis
Demodex_folliculorum
Panonychus_citri
Panonychus_ulmi
Tetranychus_cinnabarinus
Tetranychus_kanzawai
Tetranychus_pueraricola
Tetranychus_urticae
Tetranychus_truncatus
Tetranychus_ludeni
Tetranychus_phaselus
Tetranychus_malaysiensis
Epitrimerus_sabinae
Phyllocoptes_taishanensis
Allothyrus_sp
Amblyomma_cajennense
Amblyomma_triguttatum
Amblyomma_fimbriatum
Dermacentor_nitens
Rhipicephalus_australis
Rhipicephalus_microplus
Rhipicephalus_simus
Rhipicephalus_geigyi
Rhipicephalus_sanguineus
Amblyomma_sphenodont
Haemaphysalis_flava
Haemaphysalis_formosensis
Haemaphysalis_parva
Bothriocroton_concolor
Bothriocroton_undatum
Amblyomma_elaphense
Ixodes_hexagonus
Ixodes_pavlovsky
Ixodes_persulcatus
Ixodes_ricinus
Ixodes_holocyclus
Ixodes_uriae_mitochondria
Antricola_mexicanus
Carios_capensis
Ornithodoros_brasiliensis
Ornithodoros_rostratus
Ornithodoros_moubata
Ornithodoros_porcinus
Otobius_megnini
Argas_africolumbae
Argas_sp_
Argas_lagenoplastis
Argas_miniatus
Nuttalliella_namaqua
Metaseiulus_occidentalis
Phytoseiulus_persimilis
Varroa_destructo
Stylochyrus_rarior
0.5
D

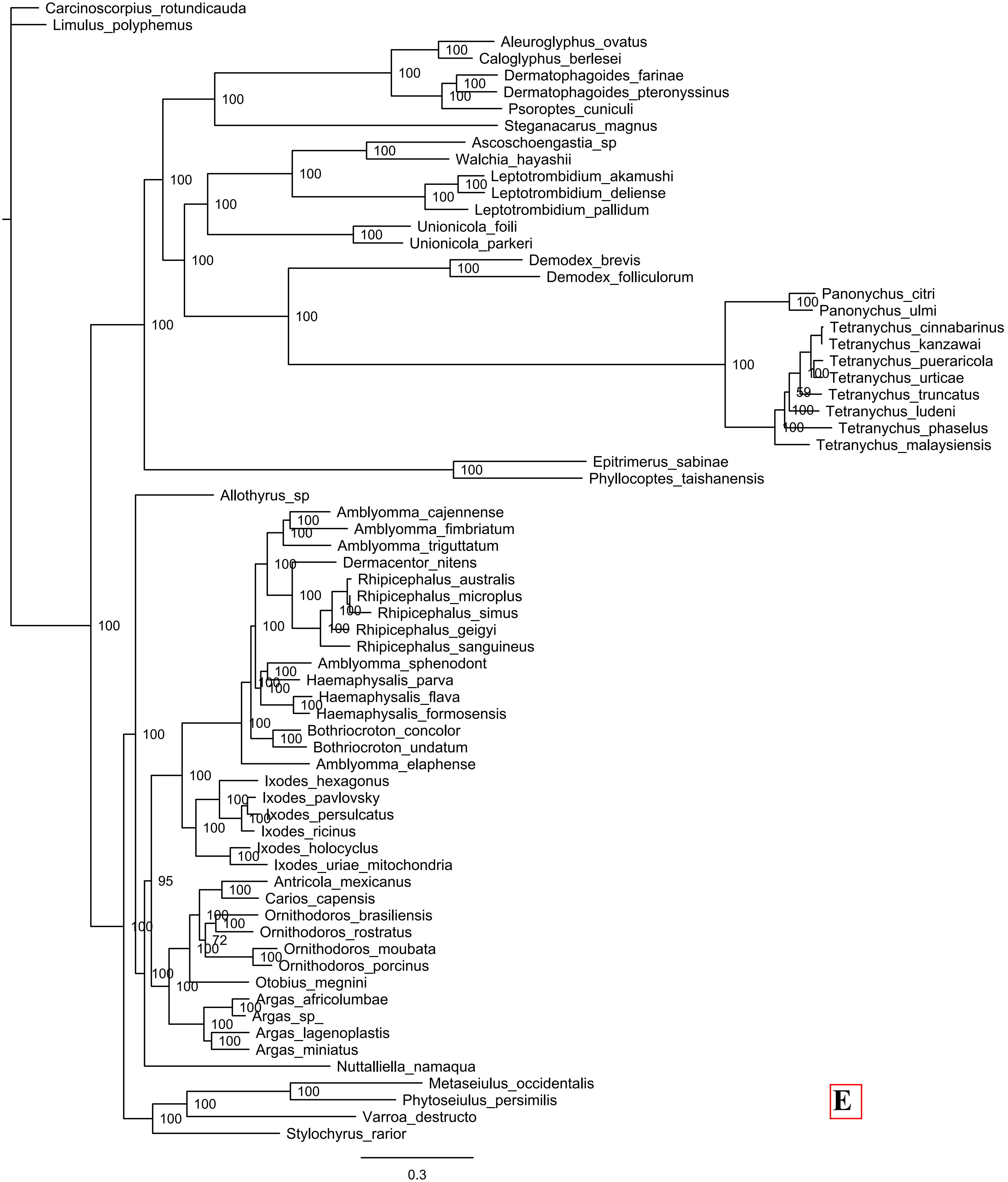

Carcinoscorpius_rotundicauda
Limulus_polyphemus
Aleuroglyphus_ovatus
Caloglyphus_berlesei
Dermatophagoides_farinae
Dermatophagoides_pteronyssinus
Psoroptes_cuniculi
Steganacarus_magnus
Ascoschoengastia_sp
Walchia_hayashii
Leptotrombidium_akamushi
Leptotrombidium_deliense
Leptotrombidium_pallidum
Unionicola_foili
Unionicola_parkeri
Demodex_brevis
Demodex_folliculorum
Panonychus_citri
Panonychus_ulmi
Tetranychus_cinnabarinus
Tetranychus_kanzawai
Tetranychus_pueraricola
Tetranychus_urticae
Tetranychus_truncatus
Tetranychus_ludeni
Tetranychus_phaselus
Tetranychus_malaysiensis
Epitrimerus_sabinae
Phyllocoptes_taishanensis
Allothyrus_sp
Amblyomma_cajennense
Amblyomma_fimbriatum
Amblyomma_triguttatum
Dermacentor_nitens
Rhipicephalus_australis
Rhipicephalus_microplus
Rhipicephalus_simus
Rhipicephalus_geigyi
Rhipicephalus_sanguineus
Amblyomma_sphenodont
Haemaphysalis_parva
Haemaphysalis_flava
Haemaphysalis_formosensis
Bothriocroton_concolor
Bothriocroton_undatum
Amblyomma_elaphense
Ixodes_hexagonus
Ixodes_pavlovsky
Ixodes_persulcatus
Ixodes_ricinus
Ixodes_holocyclus
Ixodes_uriae_mitochondria
Antricola_mexicanus
Carios_capensis
Ornithodoros_brasiliensis
Ornithodoros_rostratus
Ornithodoros_moubata
Ornithodoros_porcinus
Otobius_megnini
Argas_africolumbae
Argas_sp_
Argas_lagenoplastis
Argas_miniatus
Nuttalliella_namaqua
Metaseiulus_occidentalis
Phytoseiulus_persimilis
Varroa_destructo
Stylochyrus_rarior
0.3
E

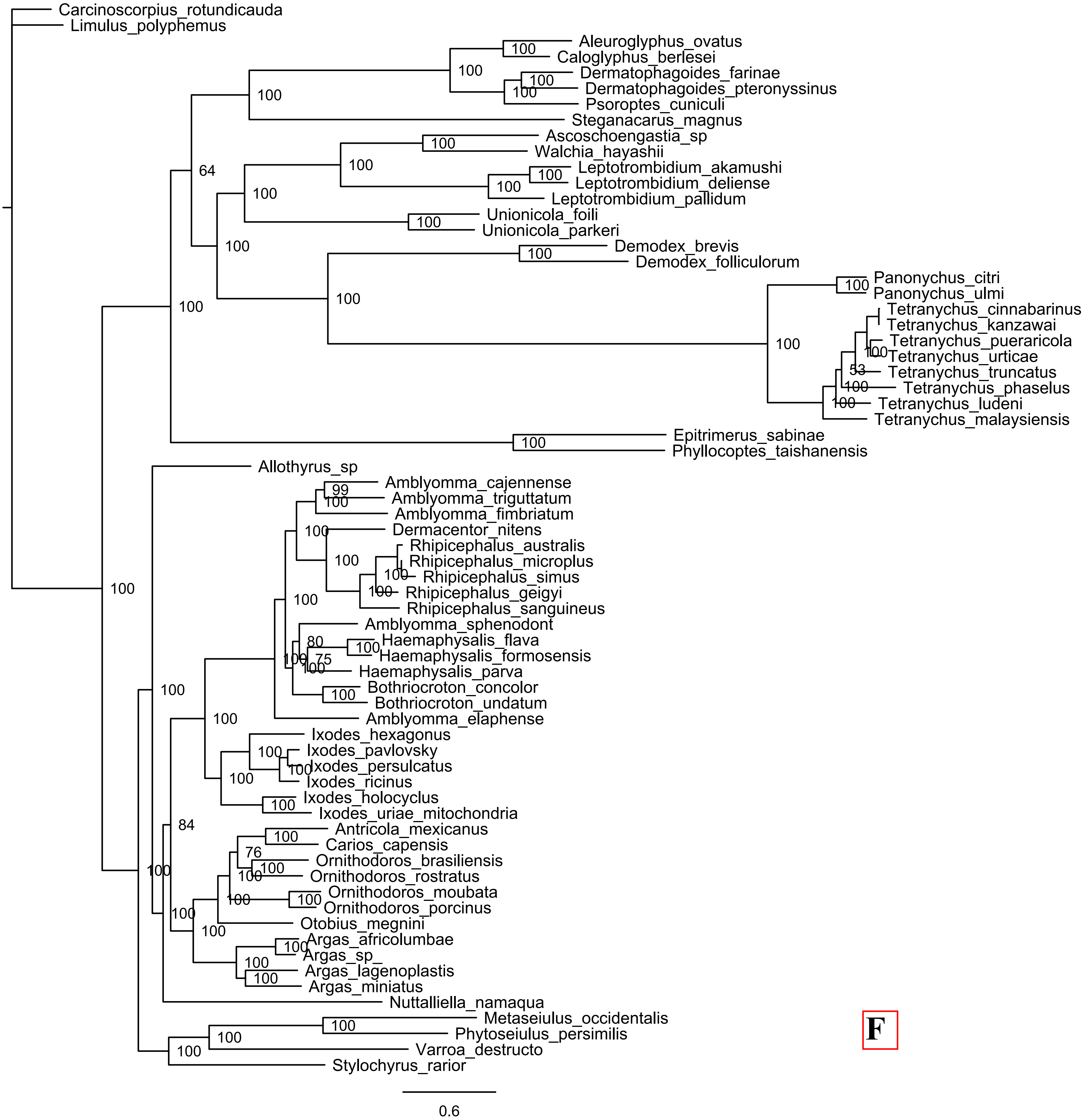
Carcinoscorpius_rotundicauda
Limulus_polyphemus
Aleuroglyphus_ovatus
Caloglyphus_berlesei
Dermatophagoides_farinae
Dermatophagoides_pteronyssinus
Psoroptes_cuniculi
Steganacarus_magnus
Ascoschoengastia_sp
Walchia_hayashii
Leptotrombidium_akamushi
Leptotrombidium_deliense
Leptotrombidium_pallidum
Unionicola_foili
Unionicola_parkeri
Demodex_brevis
Demodex_folliculorum
Panonychus_citri
Panonychus_ulmi
Tetranychus_cinnabarinus
Tetranychus_kanzawai
Tetranychus_pueraricola
Tetranychus_urticae
Tetranychus_truncatus
Tetranychus_phaselus
Tetranychus_ludeni
Tetranychus_malaysiensis
Epitrimerus_sabinae
Phyllocoptes_taishanensis
Allothyrus_sp
Amblyomma_cajennense
Amblyomma_triguttatum
Amblyomma_fimbriatum
Dermacentor_nitens
Rhipicephalus_australis
Rhipicephalus_microplus
Rhipicephalus_simus
Rhipicephalus_geigyi
Rhipicephalus_sanguineus
Amblyomma_sphenodont
Haemaphysalis_flava
Haemaphysalis_formosensis
Haemaphysalis_parva
Bothriocroton_concolor
Bothriocroton_undatum
Amblyomma_elaphense
Ixodes_hexagonus
Ixodes_pavlovsky
Ixodes_persulcatus
Ixodes_ricinus
Ixodes_holocyclus
Ixodes_uriae_mitochondria
Antricola_mexicanus
Carios_capensis
Ornithodoros_brasiliensis
Ornithodoros_rostratus
Ornithodoros_moubata
Ornithodoros_porcinus
Otobius_megnini
Argas_africolumbae
Argas_sp_
Argas_lagenoplastis
Argas_miniatus
Nuttalliella_namaqua
Metaseiulus_occidentalis
Phytoseiulus_persimilis
Varroa_destructo
Stylochyrus_rarior
F
0.6

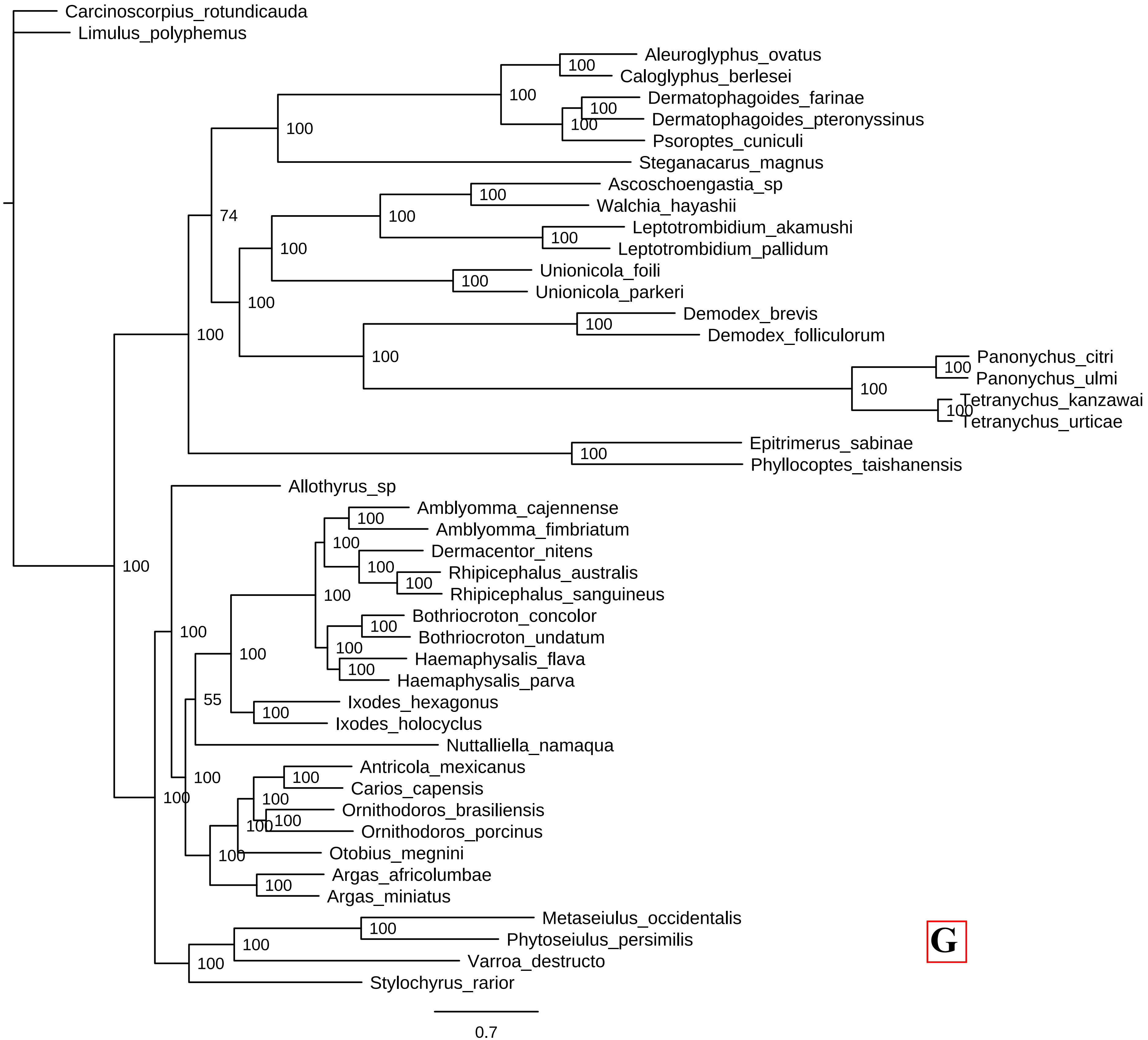

Carcinoscorpius_rotundicauda
Limulus_polyphemus
Aleuroglyphus_ovatus
Caloglyphus_berlesei
Dermatophagoides_farinae
Dermatophagoides_pteronyssinus
Psoroptes_cuniculi
Steganacarus_magnus
Ascoschoengastia_sp
Walchia_hayashii
Leptotrombidium_akamushi
Leptotrombidium_pallidum
Unionicola_foili
Unionicola_parkeri
Demodex_brevis
Demodex_folliculorum
Panonychus_citri
Panonychus_ulmi
Tetranychus_kanzawai
Tetranychus_urticae
Epitrimerus_sabinae
Phyllocoptes_taishanensis
Allothyrus_sp
Amblyomma_cajennense
Amblyomma_fimbriatum
Dermacentor_nitens
Rhipicephalus_australis
Rhipicephalus_sanguineus
Bothriocroton_concolor
Bothriocroton_undatum
Haemaphysalis_flava
Haemaphysalis_parva
Ixodes_hexagonus
Ixodes_holocyclus
Nuttalliella_namaqua
Antricola_mexicanus
Carios_capensis
Ornithodoros_brasiliensis
Ornithodoros_porcinus
Otobius_megnini
Argas_africolumbae
Argas_miniatus
Metaseiulus_occidentalis
Phytoseiulus_persimilis
Varroa_destructo
Stylochyrus_rarior
74
55
100
0.7
G

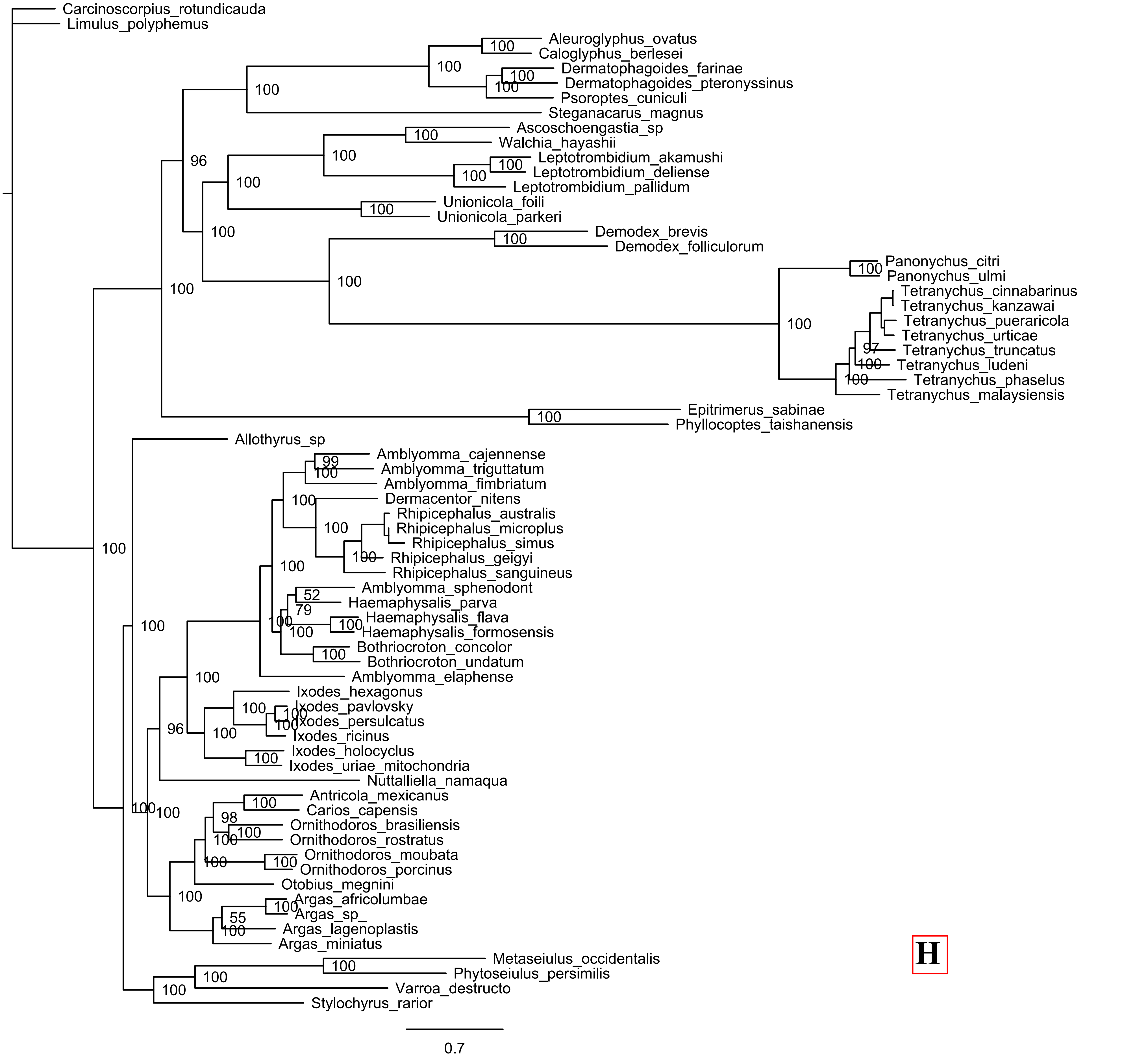
Carcinoscorpius_rotundicauda
Limulus_polyphemus
Aleuroglyphus_ovatus
Caloglyphus_berlesei
Dermatophagoides_farinae
Dermatophagoides_pteronyssinus
Psoroptes_cuniculi
Steganacarus_magnus
Ascoschoengastia_sp
Walchia_hayashii
Leptotrombidium_akamushi
Leptotrombidium_deliense
Leptotrombidium_pallidum
Unionicola_foili
Unionicola_parkeri
Demodex_brevis
Demodex_folliculorum
Panonychus_citri
Panonychus_ulmi
Tetranychus_cinnabarinus
Tetranychus_kanzawai
Tetranychus_pueraricola
Tetranychus_urticae
Tetranychus_truncatus
Tetranychus_ludeni
Tetranychus_phaselus
Tetranychus_malaysiensis
Epitrimerus_sabinae
Phyllocoptes_taishanensis
Allothyrus_sp
Amblyomma_cajennense
Amblyomma_triguttatum
Amblyomma_fimbriatum
Dermacentor_nitens
Rhipicephalus_australis
Rhipicephalus_microplus
Rhipicephalus_simus
Rhipicephalus_geigyi
Rhipicephalus_sanguineus
Amblyomma_sphenodont
Haemaphysalis_parva
Haemaphysalis_flava
Haemaphysalis_formosensis
Bothriocroton_concolor
Bothriocroton_undatum
Amblyomma_elaphense
Ixodes_hexagonus
Ixodes_pavlovsky
Ixodes_persulcatus
Ixodes_ricinus
Ixodes_holocyclus
Ixodes_uriae_mitochondria
Nuttalliella_namaqua
Antricola_mexicanus
Carios_capensis
Ornithodoros_brasiliensis
Ornithodoros_rostratus
Ornithodoros_moubata
Ornithodoros_porcinus
Otobius_megnini
Argas_africolumbae
Argas_sp_
Argas_lagenoplastis
Argas_miniatus
Metaseiulus_occidentalis
Phytoseiulus_persimilis
Varroa_destructo
Stylochyrus_rarior
0.7
H

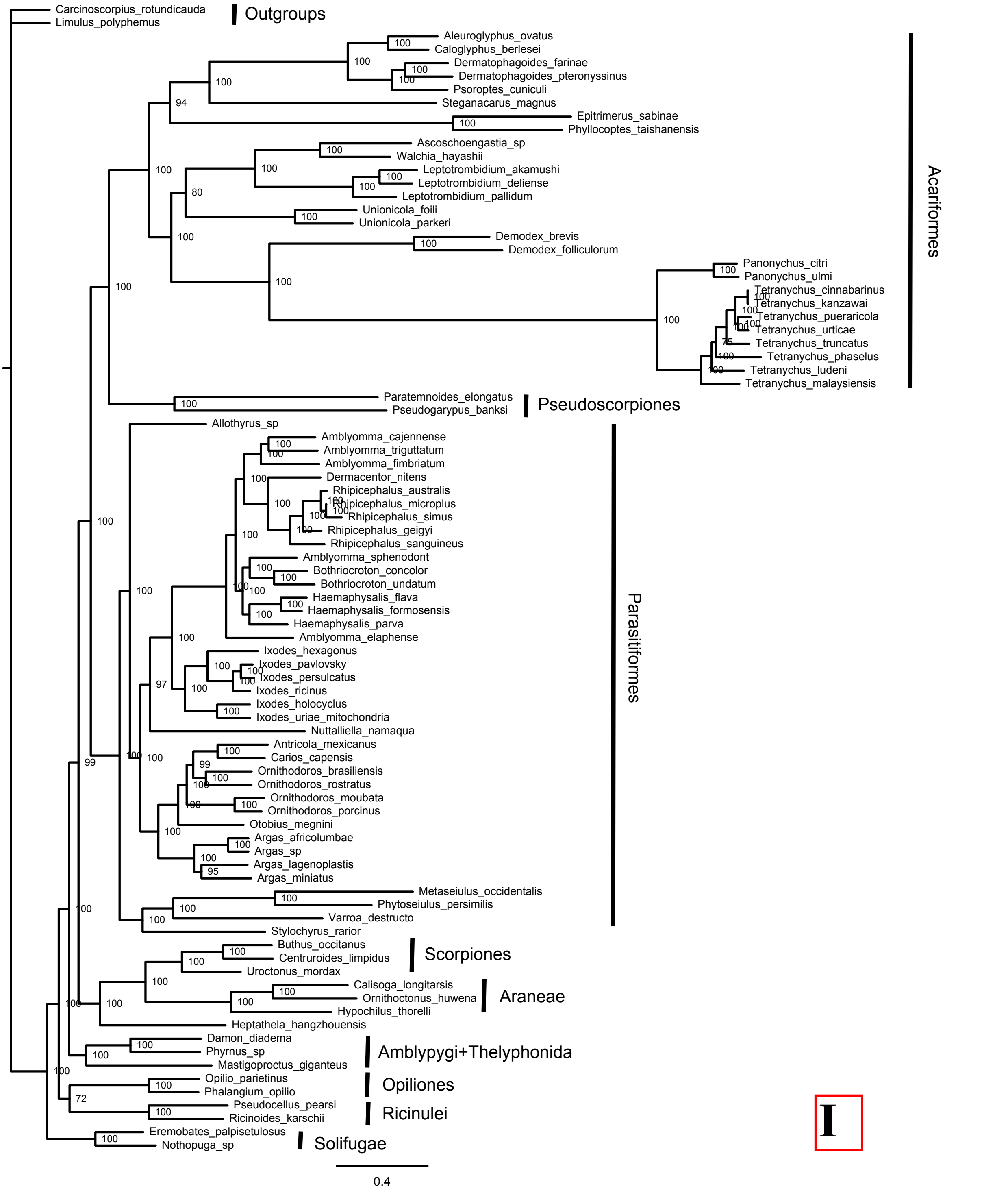

Outgroups
Carcinoscorpius_rotundicauda
Limulus_polyphemus
Acariformes
Aleuroglyphus_ovatus
Caloglyphus_berlesei
Dermatophagoides_farinae
Dermatophagoides_pteronyssinus
Psoroptes_cuniculi
Steganacarus_magnus
Epitrimerus_sabinae
Phyllocoptes_taishanensis
Ascoschoengastia_sp
Walchia_hayashii
Leptotrombidium_akamushi
Leptotrombidium_deliense
Leptotrombidium_pallidum
Unionicola_foili
Unionicola_parkeri
Demodex_brevis
Demodex_folliculorum
Panonychus_citri
Panonychus_ulmi
Tetranychus_cinnabarinus
Tetranychus_kanzawai
Tetranychus_pueraricola
Tetranychus_urticae
Tetranychus_truncatus
Tetranychus_phaselus
Tetranychus_ludeni
Tetranychus_malaysiensis
Pseudoscorpiones
Paratemnoides_elongatus
Pseudogarypus_banksi
Parasitiformes
Allothyrus_sp
Amblyomma_cajennense
Amblyomma_triguttatum
Amblyomma_fimbriatum
Dermacentor_nitens
Rhipicephalus_australis
Rhipicephalus_microplus
Rhipicephalus_simus
Rhipicephalus_geigyi
Rhipicephalus_sanguineus
Amblyomma_sphenodont
Bothriocroton_concolor
Bothriocroton_undatum
Haemaphysalis_flava
Haemaphysalis_formosensis
Haemaphysalis_parva
Amblyomma_elaphense
Ixodes_hexagonus
Ixodes_pavlovsky
Ixodes_persulcatus
Ixodes_ricinus
Ixodes_holocyclus
Ixodes_uriae_mitochondria
Nuttalliella_namaqua
Antricola_mexicanus
Carios_capensis
Ornithodoros_brasiliensis
Ornithodoros_rostratus
Ornithodoros_moubata
Ornithodoros_porcinus
Otobius_megnini
Argas_africolumbae
Argas_sp
Argas_lagenoplastis
Argas_miniatus
Metaseiulus_occidentalis
Phytoseiulus_persimilis
Varroa_destructo
Stylochyrus_rarior
Scorpiones
Buthus_occitanus
Centruroides_limpidus
Uroctonus_mordax
Araneae
Calisoga_longitarsis
Ornithoctonus_huwena
Hypochilus_thorelli
Heptathela_hangzhouensis
Amblypygi+Thelyphonida
Damon_diadema
Phrynus_sp
Mastigoproctus_giganteus
Opiliones
Opilio_parietinus
Phalangium_opilio
Ricinulei
Pseudocellus_pearsi
Ricinoides_karschii
Solifugae
Eremobates_palpisetulosus
Nothopuga_sp
0.4
I

Supplementary Table S1 D- or T-ram loss of tRNAs in the Acariformes.

**Family**	**Species**	***A***	***C***	***D***	***E***	***F***	***G***	***H***	***I***	***K***	***L_1***	***L_2***	***M***	***N***	***P***	***Q***	***R***	***S_1***	***S_2***	***T***	***V***	***W***	***Y***
Eriophyidae	*Phyllocoptes taishanensis*	├	┤	┤	┤	┤	┼	┤	├	┼	┼	┼	┼	┤	┤	┤	├	├	├	┤	┤	┼	├
	Epitrimerus sabinae	├	┤	┤	┤	┤	┼	┤	├	┼	┼	┼	┼	┤	┤	┤	├	├	├	┤	┤	┼	├
Tetranychidae	*Panonychus citri*	┤	┤	├	├	┤	┤	┤	┤	┼	┤	┼	┤	┼	┤	├	┤	├	├	┤	├	┤	┤
	Panonychus ulmi	┤	┤	├	├	┤	┤	┤	┤	┼	┤	┼	┤	┼	│	├	┤	├	├	┤	├	┤	┤
	Tetranychus truncates	┤	┤	├	├	┤	┤	┤	┤	┼	┤	┼	┤	┼	┤	│	┤	├	├	┤	├	┤	┤
	Tetranychus urticae	┤	┤	├	├	┤	┤	┤	┤	┼	┤	┼	┤	┼	┤	│	┤	├	├	┤	├	┤	┤
	Tetranycus kanzawai	┤	┤	├	├	┤	┤	┤	┤	┼	┤	┼	┤	┼	│	├	┤	├	├	┤	├	┤	┤
	Tetranycus ludeni	┤	┤	├	├	┤	┤	┤	┤	┼	┤	┼	┤	┼	│	│	┤	├	├	┤	├	┤	┤
	Tetranycus malaysiensis	┤	┤	├	├	┤	┤	┤	┤	┼	┤	┼	┤	┼	├	│	┤	│	├	┤	├	┤	┤
	Tetranycus phaselus	┤	┤	├	├	┤	┤	┤	┤	┼	┤	┼	┤	┼	│	│	┤	├	├	┤	├	┤	┤
	Tetranycus pueraricola	┤	┤	├	├	┤	┤	┤	┤	┼	┤	┼	┤	┼	│	│	┤	├	├	┤	├	┤	┤
Demodicidae	*Demodex brevis*	│	┤	│	┼	┤	┤	┤	├	┼	┤	┼	┼	┼	┤	┼	│	├	│	│	┤	┼	├
	Demodex folliculorum	│	┤	│	┼	┤	┤	┤	├	┼	┤	┼	┼	┼	┤	┼	│	├	│	│	┤	┼	├
Trombiculidae	*Leptotrombidium pallidum*	├	┤	┼	┼	┤	├	┤	├	┼	├	┤	┼	├	┤	┤	├	├	├	┤	├	┤	├
	Leptotrombidium akamushi	├	┤	┼	┼	┤	├	┤	├	┼	├	┼	┼	├	┤	┤	├	├	├	┤	├	┤	├
	Leptotrombidium deliense	├	┤	┼	┼	┤	├	┤	├	┼	├	┼	┼	├	┤	┤	├	├	├	┤	├	┤	├
	Walchia hayashii	├	┤	┼	┼	┤	├	┤	├	┼	├	┼	┼	├	┤	┼	├	├	├	┤	├	┤	├
Unionicolidae	*Unionicola foili*	├	┤	┼	┤	┤	├	┤	├	┼	┼	┼	┼	┼	┤	┼	├	├	├	┤	├	┤	┤
	Unionicola parkeri	├	┤	┼	┤	┤	├	┤	├	┼	┼	┼	┼	┼	┤	┼	├	├	├	┤	├	┤	┤
Acaridae	*Aleuroglyphus ovatus*	├	┤	┤	┤	┤	┤	┤	┤	┼	┤	┤	┤	┤	┤	┤	├	├	├	┤	├	┤	├
	Caloglyphus berlesei	├	┤	┤	┤	┤	┤	┤	┤	┼	┤	┤	┤	┤	┤	┤	├	├	├	┤	├	┤	├
Pyroglyphidae	*Dermatophagoides pteronyssinus*	├	┤	┤	┤	┤	┤	┤	┤	┼	┤	┤	┤	┤	┤	┤	├	├	├	┤	├	┤	├
	Dermatophagoides farinae	├	┤	┤	┤	┤	┤	┤	┤	┼	┤	┤	┤	┤	┤	┤	├	├	├	┤	├	┤	├
Steganacaridae	*Steganacarus magnus*	-	-	-	-	-	-	┤	-	-	-	├	-	-	┤	┤	-	-	├	-	-	┤	-

Psoroptidae	*Psoroptes cuniculi*	├	┤	┤	┤	┤	┤	┤	┤	┼	┤	┤	┤	┤	┤	┤	┤	├	├	┤	├	┤	├

"├"means D-armless, "┤" means T-armless, "│" means both T-armless and D-armless, "┼" means typical cloverleaf structure, "-" means tRNA gene loss, tRNA genes are indicated by the single letter IUPAC-IUB abbreviation for their corresponding amino acid. Unpublished data of *Ascoschoengastia* sp. was not included in this analysis.

Supplementary Table S2 PCR primers used in this study.

Primer	Sequence (5'-3')	Source
LR-J-122887	CCGGTCTGAACTCAGATCACGT	Simon et al. (1994)
LR-N-13398	CGCCTGTTTAACAAAAACAT	Simon et al. (1994)
LCO1490	GGTCAACAAATCATAAAGATATTGG	Folmer et al. (1994)
HCO2198	TAAACTTCAGGGTGACCAAAAAATCA	Folmer et al. (1994)
ECOISR3	CCGACAAATCCGTATGAAATAAGACACC	This study
E16SSR2	ACCTATTCTCACAAGAGTTTGCGACC	This study
PF2	GCAGGTCAGAATCAAGACAAACAATAACAAA	This study
PR2	TCCCTTTGATGCTAGGTGCCCACGATAT	This study
TY16sR1	TGAAATAACACAAAATAATAAACTACTT	This study
TYCB3R2	ATCGTGAAACGCTAAAGAACCAGGCT	This study
RTY16sF4	AGATTAACTTGTAGGGTCTTCTCGTCTTGCAG	This study
RTYCB3F5	TTGGCCGGGGCGATTACTATGCTTCTAACGGA	This study

Supplementary Table S3 Species used in this study.

Class	Superorder	Order	Superfamily	Family	Species	GenBank number	Size (bp)	Reference
Merostomata		Xiphosura		Limulidae	*Limulus polyphemus*	AF216203	14,985	Lavrov et al. 2000
					Carcinoscorpius rotundicauda	JX437074	15,037	Unpublished
Arachnida		Amblypygi		Phrynidae	*Phyrnus* sp.	EU520641	14,764	Masta and Boore, 2008
				Phrynichidae	*Damon diadema*	NC_011293	14,786	Unpublished
		Araneae		Nemesiidae	*Calisoga longitarsis*	EU523754	14,070	Masta and Boore, 2008
				Hypochilidae	*Hypochilus thorelli*	EU523753	13,991	Masta and Boore, 2008
				Heptathelidae	*Heptathela hangzhouensis*	AY309258	14,215	Qiu et al., 2005
				Theraphosidae	*Ornithoctonus huwena*	AY309259	13,874	Qiu et al., 2005
		Opiliones		Phalangiidae	*Phalangium opilio*	EU523757	14,968	Masta and Boore, 2008
					Opilio parietinus	NC_014700	15,400	Podsiadlowski and Fahrei, 2010
		Scorpiones		Buthidae	*Buthus occitanus*	EU523755	15,060	Masta and Boore, 2008
					Centruroides limpidus	NC_006896	14,519	Dávila et al., 2005
				Chactidae	*Uroctonus mordax*	EU523756	14,840	Masta and Boore, 2008
		Solifugae		Eremobatidae	*Eremobates palpisetulosus*	EU520642	15,083	Masta and Boore, 2008
				Ammotrechidae	*Nothopuga* sp.	NC_009984	14,985	Fahrein et al., 2007
		Thelyphonida		Thelyphonidae	*Mastigoproctus giganteus*	EU520643	14,416	Masta and Boore, 2008
		Pseudoscorpiones		Feaelloidea	*Pseudogarypus banksi*	JQ040544	16,546	Ovchinnikov and Masta, 2012
				Cheliferoidea	*Paratemnoides elongatus*	JQ040543	14,368	Ovchinnikov and Masta, 2012
		Ricinulei			*Pseudocellus pearsi*	NC_009985	15,099	Fahrein et al., 2007
				Ricinoididae	*Ricinoides karschii*	NC_023452	14,614	Unpublished
	Parasitiformes	Holothyrida	Holothyroidea	Allothyridae	*Allothyrus* sp.	KC769586	14,578	Burger *et* al. 2014
		Ixodida	Ixodoidea	Argasidae	*Antricola mexicanus*	KC769591	14,415	Burger *et* al. 2014
					Argas africolumbae	JQ665720	14,440	Mans *et al*. 2012(Mans et al.,

				2012)
	*Argas lagenoplastis**	KC769587	14,478	Burger *et* al. 2014
	Argas miniatus	KC769590	14,416	Burger *et* al. 2014
	Argas sp.*	KC769588	14,450	Burger *et* al. 2014
	Carios capensis	AB075953	14,418	Shao *et al*. 2004
	Ornithodoros brasiliensis	KC769593	14,489	Burger *et* al. 2014
	*Ornithodoros moubata**	AB073679	14,398	Shao *et al*. 2004
	Ornithodoros porcinus	AB105451	14,378	Shao *et al*. 2005
	*Ornithodoros rostratus**	KC769592	14,452	Burger *et* al. 2014
	Otobius megnini	KC769589	14,430	Burger *et* al. 2014
Ixodidae	*Amblyomma cajennense*	JX573118	14,780	Burger *et al*. 2013
	*Amblyomma elaphense**	JN863729	14,627	Burger *et al*. 2012
	Amblyomma fimbriatum	JN863729	14,705	Burger *et al*. 2012
	*Amblyomma sphenodonti**	JN863731	14,772	Burger *et al*. 2012
	*Amblyomma triguttatum**	AB113317	14,704	Shao *et al*. 2005
	Bothriocroton concolor	JN863727	14,809	Burger *et al*. 2012
	Bothriocroton undatum	JN863728	14,769	Burger *et al*. 2012
	Dermacentor nitens	KC503258	14,839	Burger *et* al. 2014
	Haemaphysalis flava	AB075954	14,686	Shao *et al*. 2004
	*Haemaphysalis formosensis**	JX573135	14,676	Burger *et al*. 2013
	Haemaphysalis parva	JX573136	14,846	Burger *et al*. 2013
	Ixodes hexagonus	AF081828	14,539	Black & Roehrdanz, 1998
	Ixodes holocyclus	AB075955	15,007	Shao *et al*. 2005
	*Ixodes pavlovskyi**	KJ000060	14,575	Unpublished
	*Ixodes persulcatus**	AB073725	14,539	Shao *et al*. 2005
	*Ixodes ricinus**	JN248424	14,566	Montagna *et al*. 2012

				*Ixodes uriae**	AB087746	15,053	Shao *et al*. 2005
				Rhipicephalus australis	KC503255	14,891	Burger *et* al. 2014
				*Rhipicephalus geigyi**	KC503263	14,948	Burger *et* al. 2014
				*Rhipicephalus microplus**	KC503260	14,903	Burger *et* al. 2014
				Rhipicephalus sanguineus	AF081829	14,710	Shao *et al*. 2004
				*Rhipicephalus simus**	KJ739594	14,929	Xu et al. 2014
			Nuttalliellidae	*Nuttalliella namaqua*	NC_019663	14,425	Mans *et al*. 2012
	Mesostigmata	Dermanyssoidea	Varroidae	*Varroa destructor*	AJ493124	16,476	Navajas *et al*. 2002
		Phytoseioidea	Phytoseiidae	*Phytoseiulus persimilis*	GQ222414	16,199	Dermauw *et al*. 2010
				Metaseiulus occidentalis	EF221760	24,961	Jeyaprakash and Hoy, 2007
		Rhodacaroidea	Ologamasidae	*Stylochyrus rarior*	GQ927176	14,900	Swafford and Bond 2009
Acariformes	Trombidiformes	Cheyletoidea	Demodicidae	*Demodex brevis*	KM114225	14,211	Palopoli *et al*. 2014
				Demodex folliculorum	KM114226	14,150	Palopoli *et al*. 2014
		Eriophyoidea	Eriophyidae	*Phyllocoptes taishanensis*	KR604967	13,475	This study
				Epitrimerus sabinae	KR604966	13,531	This study
		Tetranychoidea	Tetranychidae	*Panonychus citri*	HM189212	13,077	Yuan *et al*. 2010
				Panonychus ulmi	NC_012571	13,115	Unpublished
				*Tetranychus truncates**	KM111296	13,089	Chen *et al*. 2014
				Tetranychus urticae	NC_010526	13,103	Van Leeuwen *et al*. 2008
				Tetranycus kanzawai	KJ729017	13,091	Chen *et al*. 2014
				*Tetranycus ludeni**	KJ729018	13,064	Chen *et al*. 2014
				*Tetranycus malaysiensis**	KJ729019	13,049	Chen *et al*. 2014
				*Tetranycus phaselus**	KJ729020	13,084	Chen *et al*. 2014
				*Tetranycus pueraricola**	KJ729021	13,084	Chen *et al*. 2014
				*Tetranychus cinnabarinus**	NC_014399	13,092	Unpublished
		Trombiculoidea	Trombiculidae	*Leptotrombidium pallidum*	AB180098	16,779	Shao *et al*. 2005

			Leptotrombidium akamushi	AB194045	13,698	Shao *et al*. 2006
			*Leptotrombidium deliense**	AB194044	13,731	Shao *et al*. 2006
			Walchia hayashii	NC_010595	14,857	Unpublished
			Ascoschoengastia sp.	NC_010596	16,067	Unpublished
	Hygrobatoidea	Unionicolidae	*Unionicola foili*	EU856396	14,738	Ernsting *et al*. 2009
			Unionicola parkeri	HQ386015	14,734	Edwards *et a*l. 2011
Sarcoptiformes	Acaroidea	Acaridae	*Aleuroglyphus ovatus*	KC700022	14,328	Sun *et al*. 2014
			Caloglyphus berlesei	KF499016	14,273	Sun *et al*. 2014
	Analgoidea	Pyroglyphidae	*Dermatophagoides pteronyssinus*	EU884425	14,203	Dermauw *et al*. 2009
			Dermatophagoides farinae	NC_013184	14,266	Klimov & OConnor 2009
	Phthiracaroidea	Steganacaridae	*Steganacarus magnus*	EU935607	13,818	Domes *et al*. 2008
	Sarcoptoidea	Psoroptidae	*Psoroptes cuniculi*	KJ957822	14,247	Gu *et al*. 2014

Species with asterisk were deleted in the truncated Baysian inference.

Supplementary Table S4 Partition strategies used in this study.

Date sets	Analysis methods	Number of partitions	Models and Partition scheme
Amino acid	Bayesian	13	MTREV+I+G+F: Atp6, Atp8, Cob, Cox1, Cox2, Cox3, Nad1, Nad2, Nad3, Nad4, Nad4L, Nad5, Nad6
	ML	13	MTREV: Atp6, Atp8, Cob, Cox1, Cox2, Cox3, Nad1, Nad2, Nad3, Nad4L, Nad5, Nad6<br>JTT: Nad4
By genes	Bayesian	16	GTR+I+G: Atp6, Atp8, Cob, Cox1, Cox2, Cox3, Nad1, Nad2, Nad3, Nad4, Nad4L, Nad5, Nad6, 12S, 16S, tRNAs
	ML	16	GAMMAI: Atp6, Atp8, Cob, Cox1, Cox2, Cox3, Nad1, Nad2, Nad3, Nad4, Nad4L, Nad5, Nad6, 12S, 16S, tRNAs
By genes, exclude third codon	Bayesian	16	GTR+I+G: Atp6, Atp8, Cob, Cox1, Cox2, Cox3, Nad1, Nad2, Nad3, Nad4, Nad4L, Nad5, Nad6, 12S, 16S, tRNAs
	ML	16	GAMMAI: Atp6, Atp8, Cob, Cox1, Cox2, Cox3, Nad1, Nad2, Nad3, Nad4, Nad4L, Nad5, Nad6, 12S, 16S, tRNAs
By codon	Bayesian	6	GTR+I+G: 1st codon, 2nd codon, 3rd codon, *rrnS*, *rrnL*, tRNAs
	ML	6	GAMMAI: 1st codon, 2nd codon, 3rd codon, 12S, 16S, tRNAs
By codon, exclude third codon	Bayesian	5	GTR+I+G: 1st codon, 2nd codon, 12S, 16S, tRNAs
	ML	5	GAMMAI: 1st codon, 2nd codon, 12S, 16S, tRNAs
Nucleotide sequences by PartitionFinder	Bayesian	8	GTR+I+G: (Atp6_pos1, Cob_pos1, Cox1_pos1, Cox2_pos1, Cox3_pos1, Nad3_pos1), (Atp6_pos2, Cob_pos2, Cox1_pos2, Cox2_pos2, Cox3_pos2, Nad1_pos2, Nad2_pos2, Nad3_pos2, Nad4L_pos2, Nad5_pos2, Nad6_pos2), (Atp8_pos1, Atp8_pos2, Nad1_pos1, Nad2_pos1, Nad4L_pos1, Nad5_pos1, Nad6_pos1), (Nad4_pos1, Nad4_pos2)<br>GTR+G: (Atp6_pos3, Atp8_pos3, Cob_pos3, Cox1_pos3, Cox2_pos3, Cox3_pos3, Nad2_pos3, Nad3_pos3, Nad6_pos3), (Nad1_pos3, Nad4L_pos3, Nad4_pos3, Nad5_pos3), (12S, 16S), (tRNAs)
Nucleotide sequences by PartitionFinder, with truncated taxa	Bayesian	8	GTR+I+G: (Atp6_pos1, Cob_pos1, Cox1_pos1, Cox2_pos1, Cox3_pos1, Nad3_pos1), (Atp6_pos2, Cob_pos2, Cox1_pos2, Cox2_pos2, Cox3_pos2, Nad1_pos2, Nad2_pos2, Nad3_pos2, Nad4L_pos2, Nad5_pos2, Nad6_pos2), (Atp8_pos1, Atp8_pos2, Nad1_pos1, Nad2_pos1, Nad4L_pos1, Nad5_pos1,

			Nad6_pos1), (Nad4_pos1, Nad4_pos2)<br>GTR+G: (Atp6_pos3, Atp8_pos3, Cob_pos3, Cox1_pos3, Cox2_pos3, Cox3_pos3, Nad2_pos3, Nad3_pos3, Nad6_pos3), (Nad1_pos3, Nad4L_pos3, Nad4_pos3, Nad5_pos3), (12S, 16S), (tRNAs)
Nucleotide sequences of PCGs by PartitionFinder	Bayesian	6	GTR+I+G: (Atp6_pos1, Cob_pos1, Cox1_pos1, Cox2_pos1, Cox3_pos1, Nad3_pos1), (Atp6_pos2, Cob_pos2, Cox1_pos2, Cox2_pos2, Cox3_pos2, Nad1_pos2, Nad2_pos2, Nad3_pos2, Nad4L_pos2, Nad5_pos2, Nad6_pos2), (Atp8_pos1, Atp8_pos2, Nad1_pos1, Nad2_pos1, Nad4L_pos1, Nad5_pos1, Nad6_pos1), (Nad4_pos1, Nad4_pos2)<br>GTR+G: (Atp6_pos3, Atp8_pos3, Cob_pos3, Cox1_pos3, Cox2_pos3, Cox3_pos3, Nad2_pos3, Nad3_pos3, Nad6_pos3), (Nad1_pos3, Nad4L_pos3, Nad4_pos3, Nad5_pos3)

Supplementary Table S5 Inferred second structur of *trnC*, *trnY* and *trnV* of *Dermatophagoides pteronyssinus*.

tRNA	AA-arm		D-arm		AC-arm	V-loop	T-arm	AA-arm
trnC	ATCTCTT	AA	GTTTTAAAAATAC	A	TTAGATT**GCA**AATCTAA	AATTTT		AAGAGGTT
trnY	AGACTTT			TAAT	GAAAGCT**GTA**AACTTTC	TTTT	TTGTGCTATTATGG	AGGGTTTA
trnV	*TTAAGAG*			TTTCATTATT	TTTTGCT**TAC**AACAAAA	AATC	TTTTAA	CTCTTCTG
